# Development of a predictive model for pancreatic cancer integrating serum metabolomics and polygenic risk score

**DOI:** 10.3389/fendo.2026.1903036

**Published:** 2026-09-25

**Authors:** Xiaoran Wang, Hui Yang, Jiwen Li, Xinyu Wang, Liyan Tong, Zongze Han, Yanni Li, Man Liu, Liping Guo, Simin Zhou, Ying Ran, Jingwen Zhao, Lu Zhou

**Affiliations:** Department of Gastroenterology and Hepatology, Tianjin Medical University General Hospital, Tianjin, China

**Keywords:** metabolomics, pancreatic cancer, polygenic risk score, risk prediction, UK Biobank

## Abstract

**Background:**

Pancreatic cancer (PC) is highly aggressive and associated with extremely poor prognosis. Due to the lack of specfic symptoms in the early stage, most patients have already lost the opportunity for curative surgical resection at the time of diagnosis. Therefore, improving the early identification of individuals at elevated risk of PC is clinically important.

**Methods:**

Using data from the UK Biobank, 157,235 participants were included. Cox proportional hazards models were applied to systematically evaluate the associations of clinical biomarkers, polygenic risk scores (PRS), and 143 metabolites with PC risk, with significant metabolites identified after false discovery rate correction. Elastic net regression was then used to construct a metabolomic risk score (MRS), which was integrated with clinical biomarkers and PRS to develop multivariable prediction models. Model performance was assessed using the C-index and time-dependent area under the curve (AUC).

**Results:**

During the follow-up period, 878 incident PC cases were identified. In total, 15 clinical biomarkers and 55 metabolites were significantly associated with PC risk, with the majority of lipid- and lipoprotein-related metabolites showing inverse associations. Subsequently, 52 metabolites were selected to construct the MRS. The C-index increased from 0.722 in the base model including age and sex to 0.755 with the addition of clinical biomarkers and to 0.768 after further incorporating the PRS; the addition of the MRS yielded a C-index of 0.769, with improved risk reclassification. The integrated model showed good discrimination, with time-dependent AUCs of 0.812, 0.808, 0.768, and 0.783 at 1, 3, 5, and 10 years, respectively.

**Conclusions:**

These findings support the utility of clinical and genetic factors for PC risk assessment, while the additional predictive value of metabolomic profiling warrants further validation.

## 1 Introduction

Pancreatic cancer (PC) is a highly aggressive malignancy with an extremely poor prognosis, with a 5-year survival rate of approximately 13%, ranking among the lowest of all cancers. It has become the third leading cause of cancer-related mortality in both men and women combined, and its incidence continues to rise globally (1). The poor prognosis is largely attributable to the rapid progression of the disease and the lack of specific early symptoms, resulting in most patients being diagnosed at an advanced stage when curative surgical resection is no longer feasible (2).

Early detection is considered a key strategy to improve the prognosis of PC. However, there is currently no universally accepted and effective screening strategy for early detection, posing significant challenges for timely diagnosis (3). Existing screening modalities, such as magnetic resonance imaging (MRI) and endoscopic ultrasound (EUS), although clinically valuable, are limited by high cost and invasiveness, making them unsuitable for large-scale population screening (4). Meanwhile, carbohydrate antigen 19-9 (CA19-9), a commonly used tumor biomarker in gastrointestinal cancers, has limited sensitivity and specificity for early-stage PC, thereby restricting its value in early screening for PC (5). These limitations highlight the need for approaches that can identify individuals at increased risk of PC, thereby enabling more targeted surveillance and diagnostic evaluation.

In addition to traditional clinical risk factors, such as age, sex, smoking (6, 7), and chronic pancreatitis (8–10), genetic (11, 12) and metabolic factors (13) also play important roles in the development and progression of PC. To quantify individual genetic susceptibility, polygenic risk scores (PRS) derived from genome-wide association studies (GWAS) have been shown to be significantly associated with PC risk (14–16). Moreover, metabolic abnormalities, including obesity and diabetes, are recognized as important contributors to disease progression. Accumulating evidence suggests that metabolic reprogramming is a hallmark of PC, with dysregulated lipid metabolism being particularly prominent. PC cells can remodel their metabolic networks to enhance the utilization of multiple nutrients, including glucose, amino acids, and lipids, thereby adapting to the hypoxic and nutrient-deprived tumor microenvironment and meeting the energy and biosynthetic demands required for rapid proliferation (17).

Based on these considerations, the present study leveraged data from the UK Biobank, a large prospective cohort with extensive biomedical information, to systematically evaluate the associations of traditional clinical biomarkers, PRS, and 143 metabolites with PC risk. Furthermore, we developed Cox proportional hazards (CPH) models and assessed the incremental predictive value of integrating genetic and metabolic information into conventional risk prediction frameworks.

## 2 Materials and methods

### 2.1 Data source

The UK Biobank is a large-scale population-based prospective cohort study that enrolled over 500,000 participants aged 40–69 years, who were recruited from 22 assessment centers across the United Kingdom between 2006 and 2010. Detailed baseline information was collected, including sociodemographic characteristics, lifestyle factors, physical measurements, and medical history. In addition, the UKB integrates multi-layered biomedical data resources, encompassing genomic, proteomic, and metabolomic data, and achieves long-term follow-up through linkage to national health system electronic health records, continuously capturing information on disease incidence, hospital admissions, and mortality outcomes. Further details regarding the data resource are available at: https://www.ukbiobank.ac.uk/enable-your-research/about-our-data.

### 2.2 Study population

As shown in the flowchart (**Figure 1**), among 501,646 participants from the UK Biobank, we sequentially excluded those with discordance between self-reported and genetically inferred sex (n = 14,503), participants with a metabolomics missing rate >20% (n = 214,757), those with prevalent neoplasms at baseline (n = 32,339), and those without follow-up information (n = 82,812). A total of 157,235 participants were included in the final analysis and were divided into a training set and a validation set at a 7:3 ratio. Baseline characteristics of the two cohorts are presented in **Supplementary Table 1**.

**Figure 1:**
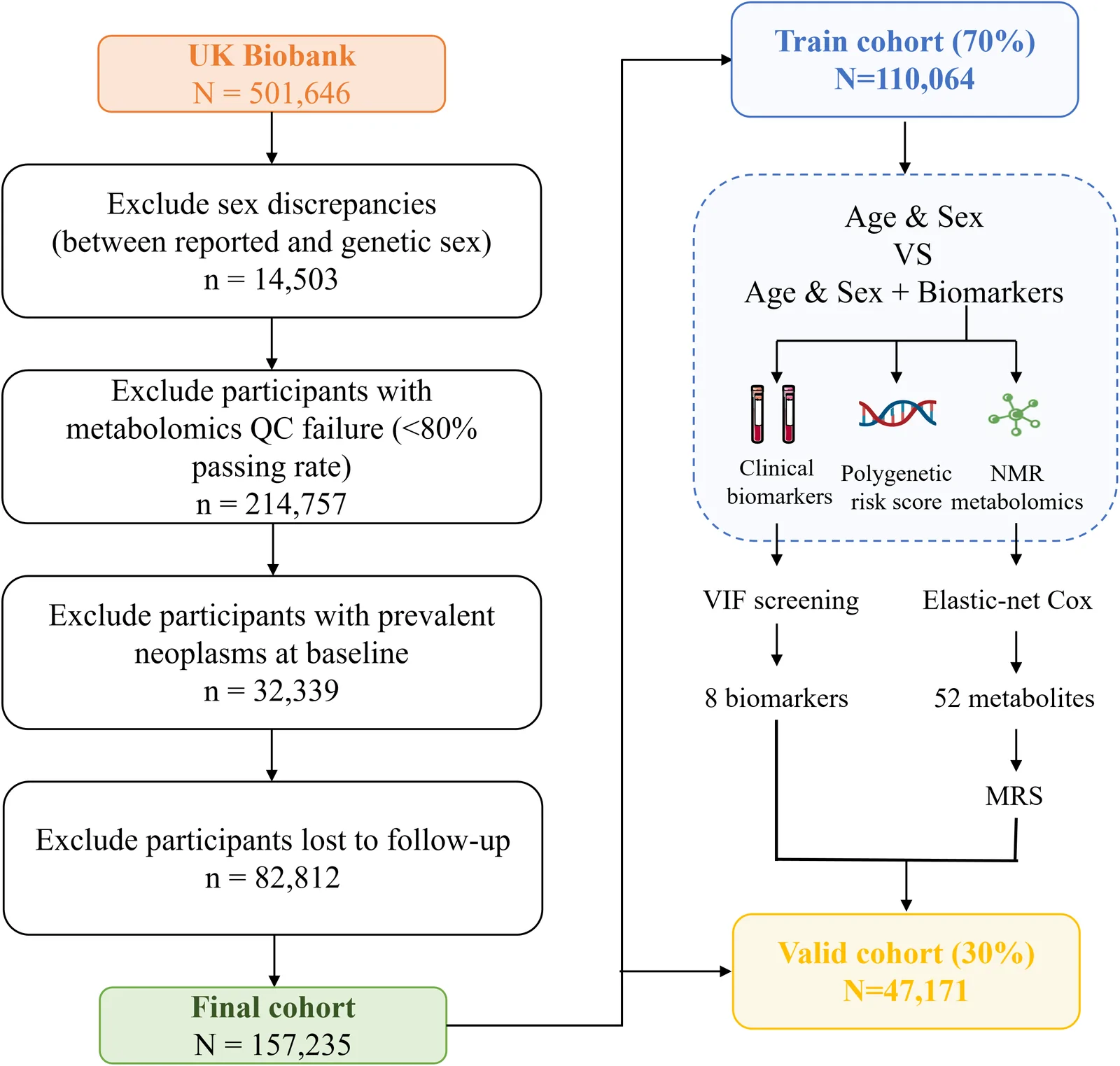
Flowchart of study population selection.

### 2.3 Ascertainment of outcome

The study outcome was PC, defined according to International Classification of Diseases (ICD)-9 code 157 and ICD-10 code C25. Outcome events were ascertained through linkage to multiple healthcare data sources, including hospital inpatient records, cancer registry data, and self-reported information from assessment centers (**Supplementary Table 2**). The time to event was defined as the interval from the baseline recruitment date to the first diagnosis of PC. For participants without PC, follow-up time was defined as the time from recruitment to the earliest occurrence of loss to follow-up, or the last available record.

### 2.4 Assessment of covariates

The covariates included in this study comprised sociodemographic characteristics, anthropometric measurements, and lifestyle factors, all of which were obtained using data-field codes from the UK Biobank. Disease history was ascertained from hospital records and self-reported data, and defined based on the timing of the first diagnosis: individuals diagnosed prior to baseline recruitment were classified as having a history of disease, while others were classified as not having a history of disease. Missing data were handled using multiple imputation implemented with the mice package in R. Predictive mean matching (PMM) was used to impute continuous variables, while logistic regression was applied for categorical variables. Five imputed datasets were generated, with all covariates, follow-up time, and outcome events included in the imputation model. Detailed definitions of all covariates are provided in **Supplementary Table 3**.

### 2.5 Construction of PRS for PC

The PRS for PC used in this study was derived from a previously published GWAS-based polygenic score and was independent of the UK Biobank cohort. Specifically, the PRS (PGS000794) was obtained from the PGS Catalog (18), which includes 22 PC-associated SNPs with inverse-variance weighted effect sizes. The SNP information and corresponding weights were obtained from the PGS Catalog. Based on individual-level genotype data, the PRS for each participant was calculated on the UK Biobank research analysis platform using PLINK by summing the weighted effect allele dosages across all SNPs (19).

### 2.6 Metabolite association analysis

In the UK Biobank, a total of 251 metabolites, primarily quantified in molar concentrations, were profiled, spanning a wide range of metabolic pathways, including lipid composition, lipoprotein subclasses, fatty acid profiles, amino acids, and glycolysis-related metabolites. To reduce redundancy and enhance biological interpretability, we selected a subset of 143 metabolites based on their absolute concentrations, excluding those derived from ratios or indirect measurements (**Supplementary Table 4**). Nevertheless, selected fatty acid ratios were retained due to their established relevance to inflammatory chronic diseases. To evaluate the associations between individual metabolites and the risk of PC, CPH models were applied. Each model included a single metabolite and was adjusted for potential confounders. Hazard ratios (HRs) and their corresponding 95% confidence intervals (CIs) were estimated. To account for multiple testing, the false discovery rate (FDR) method was employed, with an FDR-adjusted P value< 0.01 considered statistically significant (20).

### 2.7 Construction of MRS for PC

Plasma biomarker quantification was conducted using high-throughput H NMR spectroscopy. All metabolite measurements were log-transformed and subsequently standardized using Z-scores prior to analysis to improve comparability across metabolites. To reduce dimensionality and address multicollinearity among metabolites, all 143 metabolites were simultaneously entered into an elastic net penalized CPH model (21). This approach integrates both L1 (Lasso) and L2 (Ridge) penalties, enabling effective variable selection while preserving groups of highly correlated variables and improving model stability. During model training, five-fold cross-validation was used to tune hyperparameters, where λ controls the overall strength of regularization and α determines the balance between L1 and L2 penalties. The optimal combination of λ and α was selected by minimizing the cross-validated partial likelihood deviance, thereby defining the final model. Based on the optimized elastic net CPH model, metabolites with non-zero coefficients were retained as the final selected features. An MRS was then constructed using the corresponding regression coefficients (22). Specifically, for each participant, the MRS was calculated as a weighted sum of the standardized values of the selected metabolites, with weights equal to their respective regression coefficients. The MRS was calculated according to the following formula:


$$MRS\;=\;\sum_{i=1}^{n}\beta_{i}Z_{i}$$

where *n* represents the number of selected metabolites, *β_i_* represents the regression coefficient of the *i*-th metabolite derived from the optimized elastic net CPH model, and *Z_i_* represents the standardized value of the corresponding metabolite concentration for each participant. The derived MRS was subsequently standardized using Z-score transformation before incorporation into downstream prediction models.

### 2.8 Sensitivity analysis and subgroup analysis

To enhance the robustness of the primary findings, a series of sensitivity and subgroup analyses were conducted. In the sensitivity analyses, participants diagnosed with PC within the first two years of follow-up were excluded to minimize potential reverse causality between biomarkers and PC risk. Additionally, CPH models using age as the underlying time scale were applied to further assess the stability of the results. To address potential confounding from diseases that may influence metabolic levels, the CPH models were further adjusted for baseline diabetes status. Subgroup analyses were conducted by stratifying participants according to age (<55 vs. ≥55 years), sex (male vs. female), and body mass index (BMI) (<25 vs. ≥25 kg/m^2^) to evaluate potential interactions between metabolites and traditional risk factors.

### 2.9 Statistical analysis

All statistical analyses were performed using R software (version 4.5.2). For baseline characteristics, continuous variables were presented as mean (standard deviation) and compared between groups using the Student’s t-test. Categorical variables were expressed as counts (percentages) and compared using the χ2 test. CPH models were constructed using the survival package. All model development was performed exclusively in the training set. The proportional hazards assumption was assessed using Schoenfeld residuals. Among the candidate clinical variables, those significantly associated with PC risk in univariable analyses were further assessed for multicollinearity using variance inflation factors (VIF). Variables with VIF<5 were considered to have acceptable collinearity and were retained for multivariable Cox regression modeling. To evaluate model performance and the robustness of covariate effect estimates, 1,000 bootstrap resampling procedures were performed for internal validation. Based on these resamples, 95% CIs were estimated for the C-index, area under the curve (AUC), net reclassification improvement (NRI), and integrated discrimination improvement (IDI). Differences between models were considered statistically significant when the 95% CIs of the differences did not include zero.

## 3 Results

### 3.1 Baseline characteristics

A total of 157,235 participants were included in this study, with a mean age of 55.73 ± 7.88 years; 73,721 (46.89%) were males. Over a mean follow-up of 12.50 years (standard deviation, 3.38 years), 878 incident cases of PC were identified. Baseline characteristics differed significantly between individuals who developed PC and those who did not. Compared with controls, incident PC cases were more likely to be older, male, and to reside in relatively affluent areas. In terms of lifestyle factors, a higher proportion of participants in the PC group were former or current smokers, whereas alcohol consumption did not differ significantly between groups. Individuals who developed PC had significantly higher body weight, BMI, and waist circumference, while no significant difference in height was observed. In addition, the prevalence of diabetes was higher in the PC group, whereas chronic pancreatitis did not differ significantly between groups. Biochemically, the PC group exhibited higher levels of glucose, HbA1c, and liver-related biomarkers (ALT, AST, GGT, and ALP), along with elevated neutrophil counts. For lipid profiles, high-density lipoprotein (HDL) levels were significantly lower in the PC group, whereas low-density lipoprotein (LDL) levels showed no significant difference (**Table 1**).

**Table 1:** Baseline characteristics of participants stratified by incident PC.

Characteristic	Overall (N = 157,235)	Incident PC		P*
		Yes (N = 878)	No (N = 156,357)	
Follow-up time, days	4564.32 (1233.00)	3034.78 (1388.61)	4572.91 (1226.71)	<0.001
Age, years	55.73 (7.88)	61.15 (6.24)	55.70 (7.88)	<0.001
Sex				<0.001
Male	73,721 (46.89%)	467 (53.19%)	73,254 (46.85%)	
Female	83,514 (53.11%)	411 (46.81%)	83,103 (53.15%)	
Ethnicity				0.138
White	151,299 (96.22%)	836 (95.22%)	150,463 (96.23%)	
Others	5936 (3.78%)	42 (4.78%)	5894 (3.77%)	
TDI	-1.72 (2.85)	-1.34 (3.12)	-1.72 (2.85)	0.002
Physical measurements				
BMI, kg/m2	27.03 (4.58)	28.17 (4.85)	27.03 (4.58)	<0.001
Height, cm	169.24 (9.21)	169.04 (9.26)	169.24 (9.21)	0.686
Weight, kg	77.68 (15.61)	80.70 (16.20)	77.66 (15.60)	<0.001
Waist circumference	89.27 (13.18)	93.42 (13.50)	89.25 (13.17)	<0.001
Smoking status				<0.001
Never	89,093 (56.66%)	406 (46.24%)	88,687 (56.72%)	
Former	55,173 (35.09%)	340 (38.72%)	54,833 (35.07%)	
Current	12,969 (8.25%)	132 (15.03%)	12,837 (8.21%)	
Drinking status				0.007
Never or seldom	41,328 (26.28%)	255 (29.04%)	41,073 (26.27%)	
Moderate	81,015 (51.52%)	406 (46.24%)	80,609 (51.55%)	
Frequent	34,892 (22.19%)	217 (24.72%)	34,675 (22.18%)	
Diabetes mellitus				0.003
Yes	2049 (1.30%)	22 (2.51%)	2027 (1.30%)	
No	155186 (98.70%)	856 (97.49%)	154,330 (98.70%)	
Chronic pancreatitis				0.050
Yes	67 (0.04%)	2 (0.23%)	65 (0.04%)	
No	157,168 (99.96%)	876 (99.77%)	156,292 (99.96%)	
Glucose, mmol/L	5.07 (1.12)	5.35 (1.50)	5.06 (1.12)	<0.001
HbA1c, mmol/mol	35.52 (5.95)	37.74 (7.24)	35.51 (5.94)	<0.001
ALT, U/L	23.34 (13.94)	24.42 (18.38)	23.34 (13.91)	0.001
AST, U/L	25.99 (9.85)	26.80 (11.28)	25.98 (9.84)	0.009
ALP, U/L	81.51 (24.40)	85.90 (25.99)	81.49 (24.39)	<0.001
GGT, U/L	35.27 (36.10)	40.68 (39.02)	35.24 (36.08)	<0.001
LDL, mmol/L	3.57 (0.85)	3.51 (0.89)	3.57 (0.85)	0.070
HDL, mmol/L	1.46 (0.38)	1.41 (0.40)	1.46 (0.38)	<0.001
Neutrophil, 10^9^ cells/L	4.13 (1.35)	4.39 (1.45)	4.13 (1.35)	<0.001

ALP, alkaline phosphatase; ALT, alanine aminotransferase; AST, aspartate aminotransferase; BMI, body mass index; GGT, gamma-glutamyl transferase; HbA1c, glycated hemoglobin; HDL, high-density lipoprotein; LDL, low-density lipoprotein; PC, pancreatic cancer; TDI, Townsend deprivation index.

*Variables were expressed as means (SD) or number (percentages), and were compared using Student’s t-test or the χ2 test according to PC status.

### 3.2 Association of individual biomarkers with the hazard of PC

Current evidence has shown that males and older individuals have a higher risk of PC, which is consistent with the baseline characteristics observed in this study. In the base model including only sex and age, the predictive performance already reached a C-index of 0.722 (95%CI: 0.693–0.751). Building on this, we further incorporated established clinical risk factors and 143 metabolites into the training set to evaluate their incremental value in PC risk prediction. The HRs and 95% CIs for these associations are presented in **Supplementary Tables 5**, **6**. After applying a significance threshold of P< 0.01, a total of 15 clinical biomarkers and 55 metabolites were identified as significantly associated with PC risk. Similar results were observed in both the validation set and the total cohort (**Supplementary Figure 1**).

Among the clinical variables, waist circumference, BMI, body weight, HbA1c, blood glucose, smoking status, TDI, leukocyte count, neutrophil count, TG, ALP, and ALT were positively associated with PC risk, whereas HDL levels showed an inverse association. In addition, White individuals exhibited a relatively lower risk compared with other ethnic groups (**Figure 2A**).

**Figure 2:**
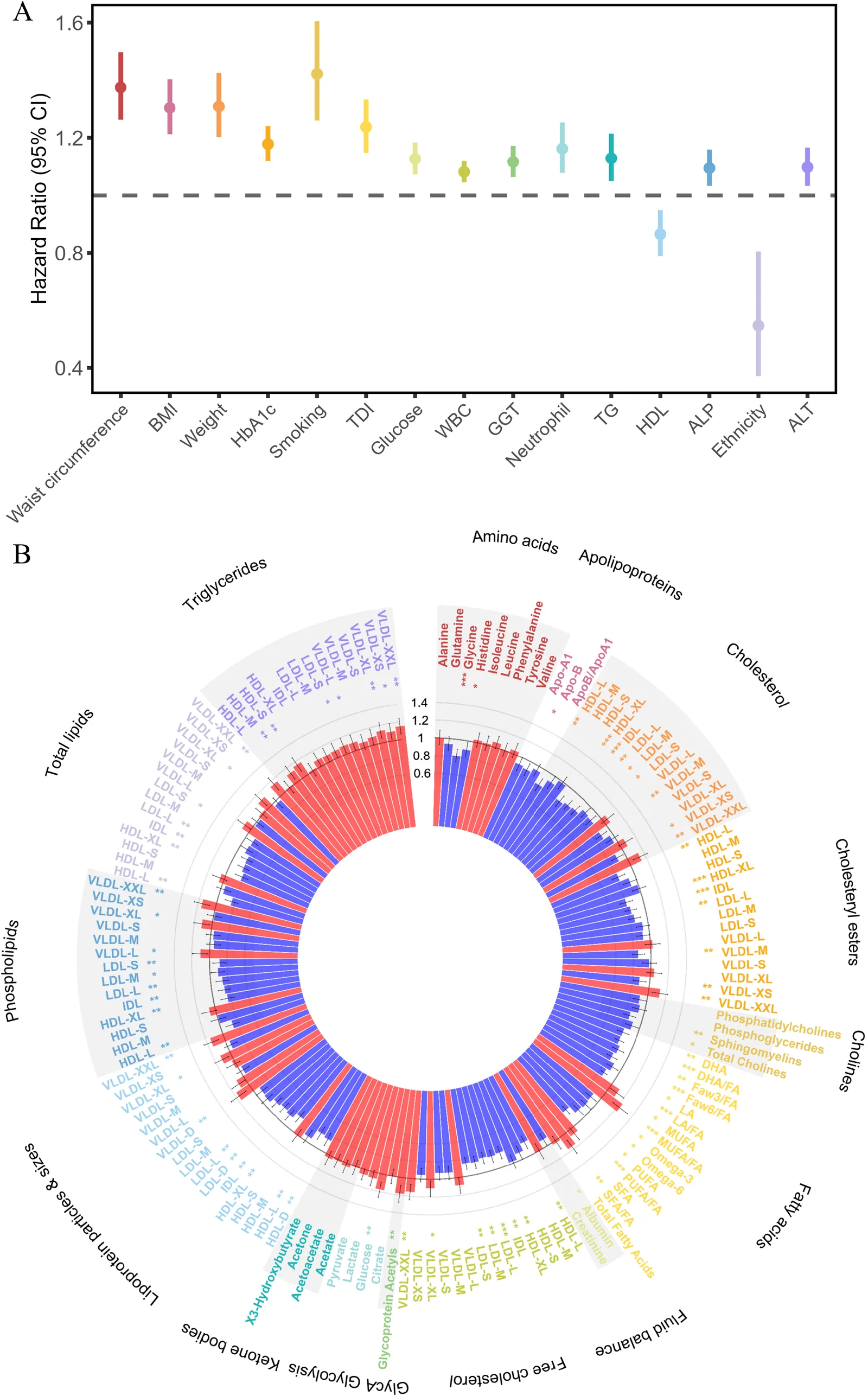
Associations of biomarkers with PC risk. **(A)** HRs and 95% CIs for the associations between clinical biomarkers (per 1-SD increase) and PC risk. HRs were adjusted for age and sex. **(B)** Associations between metabolites (per 1-SD increase on the natural log-transformed scale) and PC risk. HRs for metabolites were adjusted for age and sex. Error bars represent 95% CIs. Significant associations are indicated by *P < 0.05, **P< 0.01, ***P < 0.001, with *P*-values adjusted for the FDR. Apo-A1, apolipoprotein A1; Apo-B, apolipoprotein B; CPH, Cox proportional hazards; DHA, docosahexaenoic acid; FA, fatty acids; Faw3, omega-3 fatty acids; Faw6, omega-6 fatty acids; HDL, high-density lipoproteins; HDL-D, high-density lipoprotein particle diameter; IDL, intermediate-density lipoproteins; L, large; LA, linoleic acid; LDL, low-density lipoproteins; LDL-D, low-density lipoprotein particle diameter; M, medium; MUFA, monounsaturated fatty acids; PUFA, polyunsaturated fatty acids; S, small; SFA, saturated fatty acids; VLDL, very low-density lipoproteins; VLDL-D, very low-density lipoprotein particle diameter; XL, very large; XS, very small; XXL, extremely large.

Among the 55 significant metabolites, 40 were inversely associated with PC risk (HR< 1), predominantly reflecting lipid metabolism. These included cholesterol esters, free cholesterol, lipoprotein particles, fatty acids, cholesterol, phospholipids, and total lipids. These signals were mainly enriched in lipoprotein subclasses, particularly HDL, LDL, and intermediate-density lipoprotein particles (IDL), with the strongest associations observed in very large HDL, large HDL, and large LDL subfractions. Within fatty acid-related traits, PUFA/FA showed the strongest inverse association with PC risk (HR = 0.80, 95% CI: 0.74-0.86). Among the 15 positively associated markers, triglyceride-related traits were the most prevalent, and MUFA/FA showed the strongest positive association with PC risk (HR = 1.23, 95% CI: 1.13-1.34) (**Figure 2B**).

The associations between metabolites and PC risk remained largely unchanged after using age as the underlying time scale and further adjusting for baseline diabetes status (**Supplementary Table 6**; **Supplementary Figures 2**-**4**). However, after excluding PC cases diagnosed within the first two years of follow-up, the associations for several protein and LDL-related measures were attenuated, whereas the effects of VLDL-related biomarkers were strengthened. In subgroup analyses stratified by age, sex, and BMI, no significant interactions were observed for most metabolites (**Supplementary Table 7**; **Supplementary Figures 5**-**7**).

### 3.3 Risk prediction model

In the set of clinical biomarkers, after screening for multicollinearity, variables with strong collinearity were excluded. Ultimately, a total of eight variables statistically significant were included in the model together with age and sex, namely smoking, waist circumference, TDI, HbA1c, neutrophils, GGT, ALT, and ethnicity. Furthermore, elastic net regularization was applied to 143 metabolites, resulting in the selection of 52 biomarkers with non-zero coefficients, which were used to construct the MRS (**Supplementary Table 8**). The elastic-net cross-validation curve and coefficient path are illustrated in **Supplementary Figure 8**. On the basis of age and sex, clinical biomarkers, PRS, and the MRS were incorporated individually and in combination to build a series of models, and the relative contributions of these factors to PC prediction are shown in **Figure 3** and **Supplementary Table 9**. The proportional hazards assumption for each Cox model was evaluated using Schoenfeld residuals, and no significant violations were detected (all P > 0.05).

**Figure 3:**
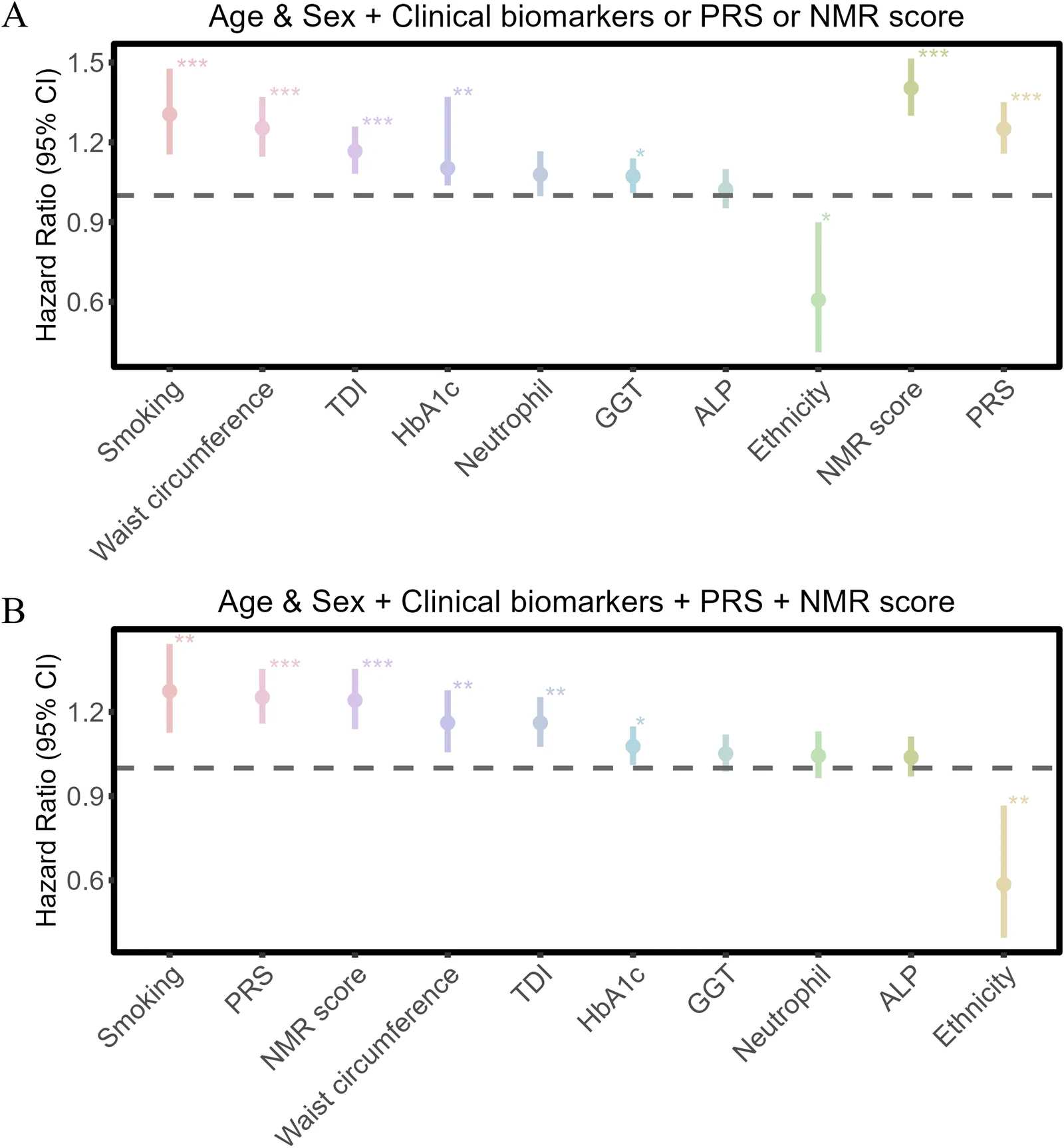
Multivariable models of clinical biomarkers, MRS, and PRS with age and sex. **(A)** Multivariable models of (1) Age & sex + clinical biomarkers, (2) Age & sex + MRS, and (3) Age & sex + PRS. **(B)** Multivariable model of Age & sex + clinical biomarkers + MRS + PRS. ALP, alkaline phosphatase; BMI, body mass index; CI, confidence interval; GGT, gamma-glutamyl transferase; HbA1c, glycated hemoglobin; MRS, metabolomic risk score; PRS, polygenic risk score; TDI, Townsend deprivation index.

In the multivariable model including age, sex, and clinical biomarkers, ethnicity showed the strongest association with PC risk (HR = 0.61, 95% CI: 0.41-0.90, P = 0.01), followed by smoking (HR = 1.31, 95% CI: 1.15-1.48, P< 0.01) and waist circumference (HR = 1.25, 95% CI: 1.15-1.37, P< 0.01). After joint modeling, the HRs of most variables were attenuated, suggesting that part of their effects may be explained by the newly included variables; however, the effect sizes of ALP, ethnicity, and PRS were increased, and most variables remained statistically significant.

### 3.4 Model performance and validation

After incorporating different types of features into the baseline model of age and sex, the predictive performance improved across all models, although the magnitude of improvement varied (**Table 2**). Among the single-domain models, which incorporated features from only one domain (clinical, PRS, or MRS), the model including eight clinical biomarkers showed the greatest improvement in discrimination, increasing the C-index from 0.722 (95% CI: 0.693-0.751) to 0.755 (95% CI: 0.728-0.783), with a ΔC-index of 0.033 (95% CI: 0.019-0.057; p<0.001). Consistent improvements were also observed in the 1-, 3-, 5-, and 10-year AUCs, along with a significant enhancement in risk reclassification. In contrast, the addition of PRS or the MRS alone resulted in relatively modest gains in model performance, with C-indices of 0.737 (95% CI: 0.709-0.765) and 0.733 (95% CI: 0.705-0.761), respectively. In the multi-domain models, those incorporating clinical features consistently outperformed the others. The model combining clinical biomarkers with PRS and the model further incorporating MRS achieved C-indices of 0.768 (95% CI: 0.741-0.795) and 0.769 (95% CI: 0.743-0.795). The incremental change in C-index between these two models was 0.001 (95% CI: 0.000-0.005; p=0.373), indicating no statistically significant improvement after adding MRS. Similar predictive performance was observed in the training set (**Supplementary Table 10**) and remained robust in the sensitivity analysis after excluding early events (**Supplementary Table 11**). Beyond discrimination, calibration analyses demonstrated good agreement between predicted and observed risks across all models (**Figure 4A**; **Supplementary Table 12**). Furthermore, the combined models consistently improved risk reclassification compared with the single-domain models (**Figure 4B**; **Supplementary Table 13**) and achieved significant risk stratification in cumulative incidence analyses (**Supplementary Figure 9**), with the corresponding cutoff values provided in **Supplementary Table 14**.

**Table 2:** Performance of risk prediction models for incident PC on the validation set.

	C-index	1-year AUC	3-year AUC	5-year AUC	10-year AUC
Age & Sex	0.722 (0.693-0.751)	0.754 (0.610-0.869)	0.768 (0.694-0.839)	0.743 (0.692-0.805)	0.740 (0.708-0.782)
Age & Sex + clinical biomarkers	0.755 (0.728-0.783)	0.784 (0.557-0.920)	0.797 (0.720-0.862)	0.764 (0.715-0.805)	0.768 (0.734-0.801)
Age & Sex + PRS	0.737 (0.709-0.765)	0.786 (0.674-0.891)	0.771 (0.698-0.845)	0.744 (0.689-0.801)	0.752 (0.718-0.792)
Age & Sex + clinical biomarkers + PRS	0.768 (0.741-0.795)	0.825 (0.685-0.938)	0.803 (0.732-0.867)	0.766 (0.720-0.821)	0.778 (0.738-0.804)
Age & Sex + MRS	0.733 (0.705-0.761)	0.705 (0.577-0.822)	0.776 (0.713-0.846)	0.752 (0.711-0.802)	0.749 (0.709-0.784)
Age & Sex + clinical biomarkers + MRS	0.756 (0.729-0.783)	0.777 (0.630-0.891)	0.805 (0.738-0.878)	0.766 (0.701-0.808)	0.770 (0.735-0.805)
Age & Sex + PRS + MRS	0.747 (0.719-0.774)	0.766 (0.681-0.857)	0.789 (0.711-0.853)	0.755 (0.708-0.805)	0.760 (0.725-0.789)
Age & Sex + clinical biomarkers + PRS + MRS	0.769 (0.743-0.795)	0.812 (0.687-0.909)	0.808 (0.752-0.858)	0.768 (0.710-0.815)	0.783 (0.749-0.808)

AUC, area under the curve; MRS, metabolomic risk score; PRS, polygenic risk score.

**Figure 4:**
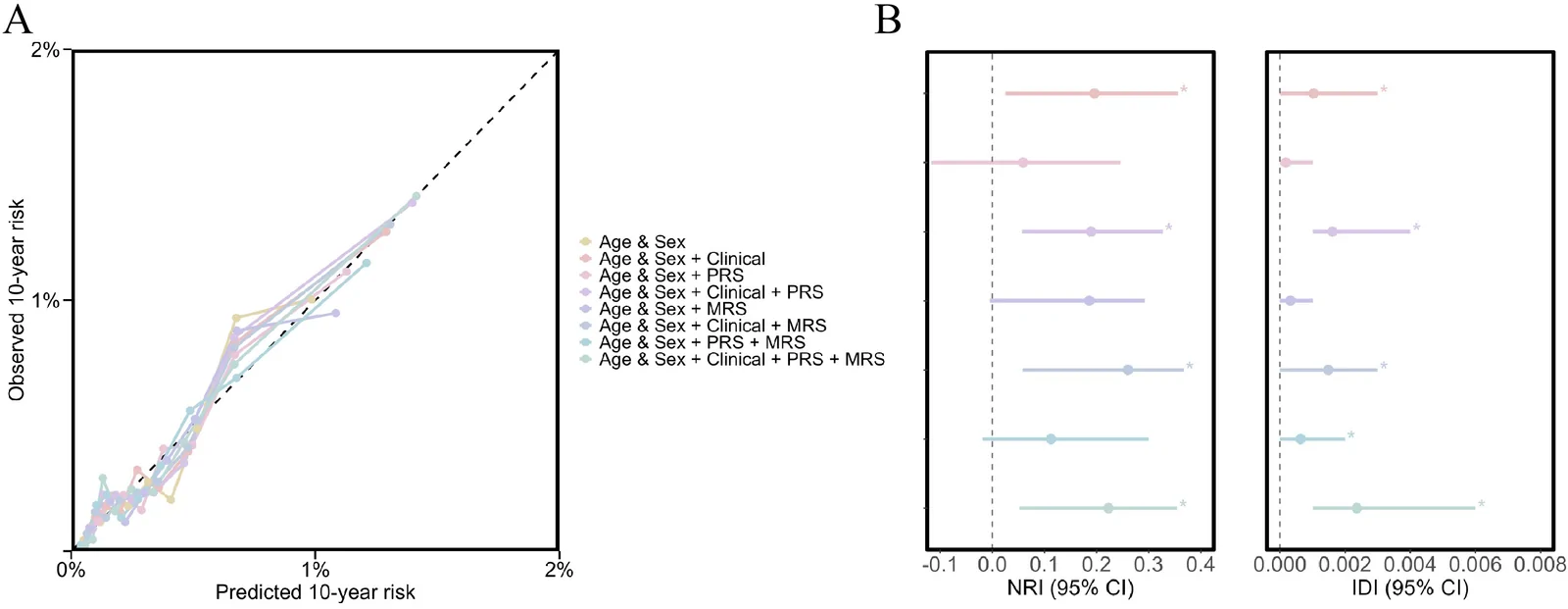
**(A)** Calibration curves of different prediction models on the validation set. **(B)** Continuous NRI and IDI between models and age & sex base model on the validation set. CI, confidence interval; IDI, integrated discrimination index; MRS, metabolomic risk score; NRI, net reclassification index; PRS, polygenic risk score.

## 4 Discussion

This study, based on the UKB database, systematically evaluated the associations of traditional clinical biomarkers, PRS, and 143 metabolites with the risk of PC. Ultimately, 15 clinical biomarkers and 55 metabolites were identified as being significantly associated with PC risk. Elastic net regression was applied to select and weight the metabolites, thereby constructing an MRS. Subsequently, clinical biomarkers, PRS, and the MRS were individually and jointly incorporated into the baseline model containing age and sex. The results demonstrated that multidimensional integration improved predictive performance to varying degrees.

The final model included eight clinical biomarkers: smoking, waist circumference, TDI, HbA1c, neutrophil count, GGT, ALT, and ethnicity. Smoking is one of the most important modifiable risk factors for PC, accounting for approximately 11%-32% of cases worldwide (23). A large meta-analysis including 45,527 PC cases showed that, compared with never-smokers, the relative risk (RR) was 1.80 (95% CI: 1.70-1.90) for current smokers and 1.20 (95% CI: 1.10-1.20) for former smokers (24). Carcinogens in tobacco, such as nicotine and nitrosamines, can enter the pancreas through the bloodstream, inducing DNA damage and gene mutations that increase tumorigenic risk. Central obesity, characterized by increased waist circumference, has been recognized as an independent risk factor for PC, even among individuals with normal BMI (25). A recent meta-analysis based on Swedish cohorts demonstrated that waist circumference (HR = 1.12) had stronger predictive power for PC risk than BMI (HR = 1.08), consistent with our findings (26). As an indicator of socioeconomic deprivation, the relationship between TDI and PC risk appears complex. On one hand, regions with higher income levels may exhibit higher PC incidence due to the accumulation of risk factors such as obesity and smoking. On the other hand, improved healthcare resources and screening accessibility may also contribute to increased detection rates (27). In addition, PC incidence is generally higher among non-White populations, which may partly be explained by higher obesity prevalence and unfavorable dietary environments. For example, minority communities often have limited access to fresh foods, while energy-dense processed foods are more readily available, thereby increasing the risk of metabolic abnormalities (28). Previous studies have demonstrated that diabetes, chronic pancreatitis, and cholelithiasis are closely associated with PC occurrence, and these conditions are often accompanied by abnormalities in biochemical and hematological parameters. Therefore, incorporating continuous laboratory indicators into predictive models may improve statistical power and enable more refined risk stratification.

Accumulating evidence suggests that systemic metabolic dysregulation—including dyslipidemia, insulin resistance, and chronic low-grade inflammation—plays a key role in pancreatic carcinogenesis (29). Epidemiological studies consistently indicate that individuals with metabolic syndrome have a significantly increased risk of PC (30–33). Mechanistically, insulin resistance is often accompanied by compensatory hyperinsulinemia, which may enhance insulin/IGF signaling and influence downstream pathways involved in cell proliferation, survival, and glucose metabolism. At the same time, PC cells exhibit substantial alterations in lipid metabolism, including increased fatty acid and cholesterol synthesis and uptake. These alterations may provide essential substrates for membrane biosynthesis and cellular energy metabolism, while lipid-derived metabolites may also modulate intracellular signaling pathways related to tumor cell proliferation and survival. In particular, dysregulation of fatty acid and cholesterol metabolism has been linked to metabolic reprogramming in PC, potentially contributing to the maintenance of a metabolically favorable environment for tumor growth (34). Collectively, these metabolic abnormalities may be related to enhanced cell proliferation, angiogenesis, and impaired apoptosis, which could contribute to pancreatic tumor initiation and progression.

In the metabolomics analysis, we identified multiple plasma metabolites significantly associated with PC, particularly lipid- and lipoprotein-related biomarkers. Higher levels of HDL and LDL particle subclasses and their lipid components, including cholesteryl esters, free cholesterol, and lipoproteins, were associated with reduced PC risk. These findings are consistent with previous studies and further support the inverse relationship between HDL and PC risk (35–37). Mechanistically, HDL plays an important role in maintaining cholesterol homeostasis and has been suggested to have potential anti-tumor effects. On one hand, HDL promotes cholesterol efflux, reducing intracellular cholesterol levels in tumor cells and thereby inhibiting proliferative signaling pathways such as AKT and MAPK while inducing apoptosis. On the other hand, HDL possesses anti-inflammatory and antioxidant properties, and reduced HDL levels may be associated with greater inflammatory activity and a tumor-promoting microenvironment, which could contribute to PC development (38). However, epidemiological evidence regarding the association between LDL and PC risk remains limited. One study reported that genetically determined higher LDL-C levels may be associated with increased PC risk, with the causal effect potentially mediated in part by BMI (39). Although our study observed protective associations between multiple LDL-related metabolites and PC risk, and these findings were consistent across both NMR-based high-throughput metabolomics and conventional biochemical indicators, subgroup analyses by BMI revealed significant interactions for most LDL-related metabolites. This suggests that the relationship between LDL and PC may be influenced by obesity status and the overall metabolic environment. Therefore, whether LDL is independently associated with PC and the underlying biological mechanisms require further validation in larger prospective cohorts across diverse populations.

Furthermore, our study highlights the potential role of fatty acid metabolic dysregulation in pancreatic carcinogenesis. We found that MUFA, SFA, and their proportions relative to total fatty acids were associated with increased PC risk, whereas PUFA and the proportion of PUFA exhibited protective effects. Previous studies similarly reported higher MUFA and SFA levels and lower PUFA levels in patients with PC compared with controls, although these associations did not reach statistical significance (40, 41). Mechanistically, SFA and MUFA may promote fatty acid β-oxidation, thereby providing ATP and other energy substrates necessary for rapid tumor cell proliferation, which may be relevant to metabolic reprogramming and the growth of PC cells. In addition, previous studies have suggested that SFA and MUFA may influence COX-2 expression and HIF-1α signaling through prostaglandin-related pathways, potentially affecting downstream targets such as VEGF and processes related to angiogenesis and tumor cell proliferation (42). In contrast, PUFA are more susceptible to lipid peroxidation and may induce ferroptosis in PC cells, thereby inhibiting tumor growth (43). These findings suggest that different fatty acid classes may be associated with PC risk through distinct metabolic and signaling pathways.

The insidious onset, rapid progression, and extremely poor prognosis of PC further underscore the importance of establishing effective risk prediction systems for early identification and disease management. Since PC diagnosis still primarily relies on imaging combined with histopathological examination, the development of stable and generalizable noninvasive predictive approaches has substantial clinical significance. In recent years, multidimensional prediction models integrating clinical, genetic, and metabolic information have emerged as an important direction in precision medicine and have demonstrated promising utility in cardiovascular diseases (44) and prostate diseases (45). Compared with traditional PC prediction models that mainly rely on demographic characteristics and clinical risk factors, our study further integrated genetic susceptibility and metabolomics data, thereby simultaneously reflecting individual genetic background and systemic metabolic status. The integration of these factors generally improved model discrimination and risk reclassification, although the incremental value varied across different information domains. Notably, adding MRS to the clinical and PRS model resulted in only a small and statistically nonsignificant increase in the C-index. Several factors may account for this finding. On the one hand, although the influence of diabetes was further considered in sensitivity analyses and the robustness of the findings was evaluated across subgroups stratified by sex, age, and BMI, some physiological and metabolic states captured by metabolomic profiles may still be correlated with information reflected by established clinical risk factors. Therefore, once clinical and genetic risk information has been incorporated into the model, the additional independent predictive information provided by MRS may be limited. On the other hand, alterations in some metabolites may primarily reflect tumor-related metabolic changes that are already present before the clinical diagnosis of PC, rather than stable risk factors capable of predicting PC over a longer time horizon. A Japanese cohort study with a median follow-up of 16.4 years included 170 incident PC cases and 340 matched healthy controls. Notably, after adjustment for potential confounding factors, none of the 12 examined metabolites was significantly associated with PC incidence when the entire follow-up period was considered. In contrast, when the analysis was further restricted to participants who were diagnosed with PC within 0–6 years after metabolite measurement, lower levels of several metabolites, including asparagine and tyrosine, were significantly associated with an increased risk of PC (46). This finding suggests that alterations in certain metabolites may reflect early metabolic changes associated with occult PC, thereby limiting their value as long-term risk predictors. Nevertheless, integrating multidimensional information may still provide a more comprehensive framework for PC risk stratification. Future studies in independent prospective cohorts, particularly those incorporating metabolomic measurements across different time windows, are warranted to better distinguish early disease-related metabolic alterations from metabolic signals that truly precede PC development and to further clarify the clinical utility of metabolomics for PC risk prediction and risk stratification.

One of the major strengths of this study is its large-scale prospective cohort design based on the UK Biobank, in which the substantial sample size and long follow-up duration enhanced the stability and reliability of risk estimation for PC. Moreover, we integrated multidimensional risk factors, including clinical, genetic, and metabolic indicators, thereby providing a relatively comprehensive framework for PC risk prediction and identification of high-risk populations. In addition, the NMR-based high-throughput metabolomics platform used in this study demonstrated excellent standardization and reproducibility, supporting its potential future clinical translation. Nevertheless, several limitations should be acknowledged. First, the UK Biobank lacks traditional PC-related predictors such as CA19-9, CEA, and family history of PC. Therefore, we were unable to evaluate the combined predictive value of these established factors with the multi-omics model, which may have limited the overall predictive performance. Second, this study included only participants from the UK Biobank, the majority of whom were of White European ancestry. Therefore, the applicability of the model to other ethnic, geographic, and clinical populations remains unverified. Further validation in independent external cohorts is warranted to evaluate the model’s generalizability and potential for broader clinical application. Finally, although the integration of PRS and metabolomics significantly improved predictive performance, the associated testing costs, complexity of data processing, and limited clinical accessibility may pose challenges for real-world implementation. Future large-scale, multicenter prospective studies involving diverse ethnic populations are therefore needed to further validate the stability, clinical applicability, and cost-effectiveness of this model, as well as to assess the practical value of combining clinical biomarkers, PRS, and metabolomics for early PC prediction and precision screening.

## 5 Conclusions

This study, based on the UKB cohort, identified multiple clinical and metabolites significantly associated with PC risk, further highlighting the critical role of lipid metabolism dysregulation in the development and progression of PC. By integrating clinical characteristics, genetic information, and metabolites, the predictive model demonstrated strong performance in risk prediction. This multidimensional model may help identify individuals at elevated risk of PC and support risk stratification; however, its clinical applicability still requires further validation in multi-ethnic and multicenter studies.

## Data Availability

The datasets analyzed in this study are not publicly available due to UK Biobank data access restrictions. Access to the data is available only to approved researchers through an application process and subject to the terms and conditions of the UK Biobank. The data used in this study were obtained under UK Biobank Application No. 535504. Requests to access these datasets should be directed to https://www.ukbiobank.ac.uk/enable-your-research/apply-for-access.
