# Supplementary material for: Development of a predictive model for pancreatic cancer integrating serum metabolomics and polygenic risk score
Source: Front Endocrinol (Lausanne). 2026 Sep 25;17:1903036. doi: 10.3389/fendo.2026.1903036 (PMC13649413; doi:10.3389/fendo.2026.1903036)
Supplement: Supplementary file 1 [file DataSheet1.docx]

**Catalogs**

Table S1 Baseline characteristics of the training and validation sets

Table S2 Data sources for ascertainment of PC outcomes

Table S3 Assessment of covariates

Table S4 Selection and distribution of metabolomics biomarkers

Table S5 Associations of clinical biomarkers with PC risk

Table S6 Associations of metabolites with PC and results of sensitivity analysis

Table S7 Subgroup analyses stratified by age, sex, and BMI

Table S8 Metabolites selected by elastic net regression with non-zero coefficients

Table S9 Relative contributions of clinical biomarkers, PRS, and the MRS in multivariable models

Table S10 Model performance in the training set

Table S11 Model performance after excluding early events

Table S12 Calibration performance of the prediction models.

Table S13 Continuous NRI and IDI between models and age & sex base model on the validation set

Table S14 Cutoff values for risk stratification

Figure S1 Associations between biomarkers and PC risk in the training set, validation set, and total cohort

Figure S2 Associations between 143 individual metabolites and PC risk, excluding PC diagnoses within the first two years of follow-up

Figure S3 Associations between 143 individual metabolites and PC risk using age as the time scale

Figure S4 Associations between 143 individual metabolites and PC risk adjusted for diabetes

Figure S5 Associations between 143 individual metabolites and PC risk stratified by age

Figure S6 Associations between 143 individual metabolites and PC risk stratified by sex

Figure S7 Associations between 143 individual metabolites and PC risk stratified by BMI

Figure S8 Best-fit Elastic Net cross-validation plot and solution path. (A) The cross-validation plot identifies the optimal parameters that minimize the cross-validation error, with α = 0.5 and λ = 0.00375857209793828. (B) The solution path offers a concise overview of all optimal solutions within the model

Figure S9 Cumulative incidence curves for risk stratification across models

Table S1 Baseline characteristics of the training and validation sets

| Characteristic* | Cohorts | |
| --- | --- | --- |
|  | Train cohort (N = 110,064) | Valid cohort (N = 47,171) |
| PC events, N | 616 (0.56%) | 262 (0.56%) |
| Follow-up time, days | 4562.89 (1234.27) | 4567.67 (1230.05) |
| Age, years | 55.75 (7.87) | 55.68 (7.90) |
| Sex |  |  |
| Male | 51,574 (46.86%) | 22,147 (46.95%) |
| Female | 58,490 (53.14%) | 25,024 (53.05%) |
| Ethnicity |  |  |
| White | 105,994 (96.30%) | 45,305 (96.04%) |
| Others | 4070 (3.70%) | 1866 (3.96%) |
| TDI | -1.72 (2.86) | -1.72 (2.84) |
| Physical measurements |  |  |
| BMI, kg/m2 | 27.03 (4.57) | 27.04 (4.61) |
| Height, cm | 169.22 (9.20) | 169.28 (9.21) |
| Weight, kg | 77.66 (15.59) | 77.71 (15.65) |
| Waist circumference | 89.27 (13.17) | 89.27 (13.19) |
| Smoking status |  |  |
| Never | 62,440 (56.73%) | 26,653 (56.50%) |
| Former | 38,525 (35.00%) | 16,648 (35.29%) |
| Current | 9099 (8.27%) | 3870 (8.2%) |
| Drinking status |  |  |
| Never or seldom | 28,873 (26.23%) | 12,455 (26.4%) |
| Moderate | 56,672 (51.49%) | 24,343 (51.61%) |
| Frequent | 24,519 (22.28%) | 10,373 (21.99%) |
| Diabetes mellitus |  |  |
| Yes | 1440 (1.31%) | 609 (1.29%) |
| No | 108,624 (98.69%) | 46,562 (98.71%) |
| Chronic pancreatitis |  |  |
| Yes | 48 (0.04%) | 19 (0.04%) |
| No | 110,016 (99.96%) | 47,152 (99.96%) |
| Glucose, mmol/L | 5.07 (1.13) | 5.08 (1.11) |
| HbA1c, mmol/mol | 35.53 (5.91) | 35.52 (6.03) |
| ALT, U/L | 20.05 (15.35-27.22) | 19.99 (15.32-27.08) |
| AST, U/L | 24.30 (20.90-28.70) | 24.30 (20.90-28.70) |
| ALP, U/L | 78.55 (65.90-93.50) | 78.70 (65.70-93.40) |
| GGT, U/L | 25.50 (18.00-39.30) | 25.30 (18.00-38.80) |
| LDL, mmol/L | 3.57 (0.85) | 3.56 (0.85) |
| HDL, mmol/L | 1.46 (0.38) | 1.46 (0.38) |
| Neutrophil, 10^9^ cells/L | 4.13 (1.35) | 4.13 (1.35) |

ALP, alkaline phosphatase; ALT, alanine aminotransferase; AST, aspartate aminotransferase; BMI, body mass index; GGT, gamma-glutamyl transferase; HbA1c, glycated hemoglobin; HDL, high-density lipoprotein; LDL, low-density lipoprotein; PC, pancreatic cancer; TDI, Townsend deprivation index

*Variables were expressed as means (SD) or number (percentages).

Table S2 Data sources for ascertainment of PC outcomes

| Source | Data field |
| --- | --- |
| Hospital inpatient | 41270, 41271 |
| Cancer register | 40006, 40013 |
| Self reported | 20001 |
| First occurance | 41280, 41281, 40005, 84 |

Table S3 Assessment of covariates

| Variable | Data field | Notes | Data type |
| --- | --- | --- | --- |
| Age | 21022 | Age at recruitment | Continuous, years |
| Sex | 31 | Male, female | Binary |
| Ethnicity | 21000 | White, non-white | Binary |
| Blood type | 23165 | Blood type O, non-O | Binary |
| TDI | 22189 | Townsend deprivation index at recruitment | Continuous |
| BMI | 21001 | Body mass index | Continuous, kg/m2 |
| Height | 12144 |  | Continuous, cm |
| Weight | 21002 |  | Continuous, kg |
| Waist circumference | 48 |  | Continuous, cm |
| Smoking status | 20116 | Never, previous, current | Tertiary |
| Drinking status | 20117 | Never, previous, current | Tertiary |
| Sleep duration | 1160 |  | Continuous, hours/day |
| Summed MET minutes | 22040 | Summed MET minutes per week for all activity | Continuous, minutes/week |
| Diabetes mellitus | 41270, 41271, 20001 | ICD10:E10-E14; ICD9:250 | Binary |
| Chronic pancreatitis | 41270, 41271, 20001 | ICD10:K86.0, K86.1; ICD9:5771 | Binary |
| Ulcerative colitis | 41270, 41271, 20001 | ICD10:K51; ICD9:556 | Binary |
| Glucose | 30740 |  | Continuous, mmol/L |
| HbA1c | 30750 | Glycated hemoglobin | Continuous, mmol/mol |
| ALT | 30620 | Alanine aminotransferase | Continuous, U/L |
| AST | 30650 | Aspartate aminotransferase | Continuous, U/L |
| ALP | 30610 | Alkaline phosphatase | Continuous, U/L |
| GGT | 30730 | Gamma glutamyltransferase | Continuous, U/L |
| TG | 30870 | Triglycerides | Continuous, mmol/L |
| Cholesterol | 30690 |  | Continuous, mmol/L |
| TBIL | 30840 | Total bilirubin | Continuous, umol/L |
| HDL | 30760 | HDL Cholesterol | Continuous, mmol/L |
| LDL | 30780 | LDL Cholesterol | Continuous, mmol/L |
| Neutrophil | 30140 |  | Continuous, 10^9 cells/Litre |
| Lymphocyte | 30120 |  | Continuous, 10^9 cells/Litre |
| WBC | 30000 | White blood cell | Continuous, 10^9 cells/Litre |
| PLT | 30080 | Platelet | Continuous, 10^9 cells/Litre |

Table S4 Selection and distribution of metabolomics biomarkers

| Data field | Variable | Group | Mean | SD |
| --- | --- | --- | --- | --- |
| 23475 | Acetate | Ketone bodies | 0.017 | 0.028 |
| 23476 | Acetoacetate | Ketone bodies | 0.013 | 0.012 |
| 23477 | Acetone | Ketone bodies | 0.014 | 0.005 |
| 23460 | Alanine | Amino acids | 0.297 | 0.078 |
| 23479 | Albumin | Albumin | 39.548 | 3.341 |
| 23441 | ApoB/ApoA1 | Apolipoproteins | 0.594 | 0.164 |
| 23440 | Apolipoprotein A1 | Apolipoproteins | 1.468 | 0.244 |
| 23439 | Apolipoprotein B | Apolipoproteins | 0.851 | 0.197 |
| 23433 | Average Diameter for HDL Particles | Lipoprotein particles & sizes | 9.648 | 0.207 |
| 23432 | Average Diameter for LDL Particles | Lipoprotein particles & sizes | 23.929 | 0.088 |
| 23431 | Average Diameter for VLDL Particles | Lipoprotein particles & sizes | 38.581 | 1.259 |
| 23484 | Cholesterol in Chylomicrons and Extremely Large VLDL | Cholesterol | 0.053 | 0.042 |
| 23526 | Cholesterol in IDL | Cholesterol | 0.846 | 0.210 |
| 23561 | Cholesterol in Large HDL | Cholesterol | 0.304 | 0.171 |
| 23533 | Cholesterol in Large LDL | Cholesterol | 1.146 | 0.280 |
| 23498 | Cholesterol in Large VLDL | Cholesterol | 0.097 | 0.048 |
| 23568 | Cholesterol in Medium HDL | Cholesterol | 0.504 | 0.123 |
| 23540 | Cholesterol in Medium LDL | Cholesterol | 0.429 | 0.116 |
| 23505 | Cholesterol in Medium VLDL | Cholesterol | 0.172 | 0.065 |
| 23575 | Cholesterol in Small HDL | Cholesterol | 0.449 | 0.061 |
| 23547 | Cholesterol in Small LDL | Cholesterol | 0.182 | 0.044 |
| 23512 | Cholesterol in Small VLDL | Cholesterol | 0.157 | 0.051 |
| 23554 | Cholesterol in Very Large HDL | Cholesterol | 0.083 | 0.035 |
| 23491 | Cholesterol in Very Large VLDL | Cholesterol | 0.053 | 0.029 |
| 23519 | Cholesterol in Very Small VLDL | Cholesterol | 0.185 | 0.049 |
| 23485 | Cholesteryl Esters in Chylomicrons and Extremely Large VLDL | Cholesteryl esters | 0.030 | 0.023 |
| 23418 | Cholesteryl Esters in IDL | Cholesteryl esters | 0.625 | 0.156 |
| 23562 | Cholesteryl Esters in Large HDL | Cholesteryl esters | 0.235 | 0.133 |
| 23534 | Cholesteryl Esters in Large LDL | Cholesteryl esters | 0.847 | 0.207 |
| 23499 | Cholesteryl Esters in Large VLDL | Cholesteryl esters | 0.051 | 0.024 |
| 23569 | Cholesteryl Esters in Medium HDL | Cholesteryl esters | 0.415 | 0.099 |
| 23541 | Cholesteryl Esters in Medium LDL | Cholesteryl esters | 0.308 | 0.087 |
| 23506 | Cholesteryl Esters in Medium VLDL | Cholesteryl esters | 0.093 | 0.038 |
| 23576 | Cholesteryl Esters in Small HDL | Cholesteryl esters | 0.333 | 0.047 |
| 23548 | Cholesteryl Esters in Small LDL | Cholesteryl esters | 0.132 | 0.033 |
| 23513 | Cholesteryl Esters in Small VLDL | Cholesteryl esters | 0.098 | 0.033 |
| 23555 | Cholesteryl Esters in Very Large HDL | Cholesteryl esters | 0.059 | 0.028 |
| 23492 | Cholesteryl Esters in Very Large VLDL | Cholesteryl esters | 0.030 | 0.015 |
| 23520 | Cholesteryl Esters in Very Small VLDL | Cholesteryl esters | 0.127 | 0.035 |
| 23473 | Citrate | Glycolysis | 0.065 | 0.013 |
| 23481 | Concentration of Chylomicrons and Extremely Large VLDL Particles | Lipoprotein particles & sizes | 0.000 | 0.000 |
| 23523 | Concentration of IDL Particles | Lipoprotein particles & sizes | 0.000 | 0.000 |
| 23558 | Concentration of Large HDL Particles | Lipoprotein particles & sizes | 0.001 | 0.001 |
| 23530 | Concentration of Large LDL Particles | Lipoprotein particles & sizes | 0.001 | 0.000 |
| 23495 | Concentration of Large VLDL Particles | Lipoprotein particles & sizes | 0.000 | 0.000 |
| 23565 | Concentration of Medium HDL Particles | Lipoprotein particles & sizes | 0.004 | 0.001 |
| 23537 | Concentration of Medium LDL Particles | Lipoprotein particles & sizes | 0.000 | 0.000 |
| 23502 | Concentration of Medium VLDL Particles | Lipoprotein particles & sizes | 0.000 | 0.000 |
| 23572 | Concentration of Small HDL Particles | Lipoprotein particles & sizes | 0.010 | 0.001 |
| 23544 | Concentration of Small LDL Particles | Lipoprotein particles & sizes | 0.000 | 0.000 |
| 23509 | Concentration of Small VLDL Particles | Lipoprotein particles & sizes | 0.000 | 0.000 |
| 23551 | Concentration of Very Large HDL Particles | Lipoprotein particles & sizes | 0.000 | 0.000 |
| 23488 | Concentration of Very Large VLDL Particles | Lipoprotein particles & sizes | 0.000 | 0.000 |
| 23516 | Concentration of Very Small VLDL Particles | Lipoprotein particles & sizes | 0.000 | 0.000 |
| 23478 | Creatinine | Fluid balance | 0.067 | 0.014 |
|  | DHA/FA | Fatty acids | 0.020 | 0.007 |
| 23450 | Docosahexaenoic Acid | Fatty acids | 0.240 | 0.084 |
|  | Faw3/FA | Fatty acids | 0.044 | 0.016 |
|  | Faw6/FA | Fatty acids | 0.381 | 0.036 |
| 23486 | Free Cholesterol in Chylomicrons and Extremely Large VLDL | Free cholesterol | 0.024 | 0.019 |
| 23528 | Free Cholesterol in IDL | Free cholesterol | 0.221 | 0.055 |
| 23563 | Free Cholesterol in Large HDL | Free cholesterol | 0.069 | 0.038 |
| 23535 | Free Cholesterol in Large LDL | Free cholesterol | 0.299 | 0.076 |
| 23500 | Free Cholesterol in Large VLDL | Free cholesterol | 0.046 | 0.025 |
| 23570 | Free Cholesterol in Medium HDL | Free cholesterol | 0.089 | 0.025 |
| 23542 | Free Cholesterol in Medium LDL | Free cholesterol | 0.121 | 0.032 |
| 23507 | Free Cholesterol in Medium VLDL | Free cholesterol | 0.079 | 0.029 |
| 23577 | Free Cholesterol in Small HDL | Free cholesterol | 0.116 | 0.016 |
| 23549 | Free Cholesterol in Small LDL | Free cholesterol | 0.050 | 0.012 |
| 23514 | Free Cholesterol in Small VLDL | Free cholesterol | 0.059 | 0.019 |
| 23556 | Free Cholesterol in Very Large HDL | Free cholesterol | 0.023 | 0.007 |
| 23493 | Free Cholesterol in Very Large VLDL | Free cholesterol | 0.023 | 0.014 |
| 23521 | Free Cholesterol in Very Small VLDL | Free cholesterol | 0.058 | 0.015 |
| 23470 | Glucose | Glycolysis | 3.635 | 1.081 |
| 23461 | Glutamine | Amino acids | 0.552 | 0.084 |
| 23462 | Glycine | Amino acids | 0.172 | 0.067 |
| 23480 | Glycoprotein Acetyls | GlycA | 0.800 | 0.117 |
| 23463 | Histidine | Amino acids | 0.066 | 0.011 |
| 23465 | Isoleucine | Amino acids | 0.051 | 0.018 |
|  | LA/FA | Fatty acids | 0.292 | 0.034 |
| 23471 | Lactate | Glycolysis | 3.946 | 1.110 |
| 23466 | Leucine | Amino acids | 0.104 | 0.029 |
| 23449 | Linoleic Acid | Fatty acids | 3.472 | 0.681 |
| 23447 | Monounsaturated Fatty Acids | Fatty acids | 2.854 | 0.814 |
|  | MUFA/FA | Fatty acids | 0.235 | 0.026 |
| 23444 | Omega-3 Fatty Acids | Fatty acids | 0.535 | 0.222 |
| 23445 | Omega-6 Fatty Acids | Fatty acids | 4.509 | 0.681 |
| 23468 | Phenylalanine | Amino acids | 0.047 | 0.011 |
| 23437 | Phosphatidylcholines | Cholines | 2.112 | 0.373 |
| 23434 | Phosphoglycerides | Cholines | 2.294 | 0.396 |
| 23483 | Phospholipids in Chylomicrons and Extremely Large VLDL | Phospholipids | 0.033 | 0.031 |
| 23525 | Phospholipids in IDL | Phospholipids | 0.294 | 0.065 |
| 23560 | Phospholipids in Large HDL | Phospholipids | 0.330 | 0.155 |
| 23532 | Phospholipids in Large LDL | Phospholipids | 0.360 | 0.081 |
| 23497 | Phospholipids in Large VLDL | Phospholipids | 0.066 | 0.040 |
| 23567 | Phospholipids in Medium HDL | Phospholipids | 0.492 | 0.100 |
| 23539 | Phospholipids in Medium LDL | Phospholipids | 0.163 | 0.042 |
| 23504 | Phospholipids in Medium VLDL | Phospholipids | 0.129 | 0.047 |
| 23574 | Phospholipids in Small HDL | Phospholipids | 0.666 | 0.092 |
| 23546 | Phospholipids in Small LDL | Phospholipids | 0.089 | 0.019 |
| 23511 | Phospholipids in Small VLDL | Phospholipids | 0.098 | 0.030 |
| 23553 | Phospholipids in Very Large HDL | Phospholipids | 0.079 | 0.045 |
| 23490 | Phospholipids in Very Large VLDL | Phospholipids | 0.038 | 0.026 |
| 23518 | Phospholipids in Very Small VLDL | Phospholipids | 0.105 | 0.026 |
| 23446 | Polyunsaturated Fatty Acids | Fatty acids | 5.043 | 0.804 |
|  | PUFA/FA | Fatty acids | 0.425 | 0.037 |
| 23472 | Pyruvate | Glycolysis | 0.081 | 0.033 |
| 23448 | Saturated Fatty Acids | Fatty acids | 4.087 | 0.944 |
|  | SFA/FA | Fatty acids | 0.339 | 0.020 |
| 23438 | Sphingomyelins | Cholines | 0.453 | 0.072 |
| 23436 | Total Cholines | Cholines | 2.580 | 0.410 |
| 23442 | Total Fatty Acids | Total lipids | 11.986 | 2.380 |
| 23482 | Total Lipids in Chylomicrons and Extremely Large VLDL | Total lipids | 0.213 | 0.199 |
| 23524 | Total Lipids in IDL | Total lipids | 1.240 | 0.284 |
| 23559 | Total Lipids in Large HDL | Total lipids | 0.664 | 0.329 |
| 23531 | Total Lipids in Large LDL | Total lipids | 1.603 | 0.373 |
| 23496 | Total Lipids in Large VLDL | Total lipids | 0.330 | 0.179 |
| 23566 | Total Lipids in Medium HDL | Total lipids | 1.049 | 0.225 |
| 23538 | Total Lipids in Medium LDL | Total lipids | 0.625 | 0.164 |
| 23503 | Total Lipids in Medium VLDL | Total lipids | 0.578 | 0.207 |
| 23573 | Total Lipids in Small HDL | Total lipids | 1.168 | 0.157 |
| 23545 | Total Lipids in Small LDL | Total lipids | 0.287 | 0.065 |
| 23510 | Total Lipids in Small VLDL | Total lipids | 0.413 | 0.132 |
| 23552 | Total Lipids in Very Large HDL | Total lipids | 0.169 | 0.081 |
| 23489 | Total Lipids in Very Large VLDL | Total lipids | 0.200 | 0.134 |
| 23517 | Total Lipids in Very Small VLDL | Total lipids | 0.358 | 0.088 |
| 23487 | Triglycerides in Chylomicrons and Extremely Large VLDL | Total lipids | 0.127 | 0.128 |
| 23529 | Triglycerides in IDL | Triglycerides | 0.100 | 0.026 |
| 23564 | Triglycerides in Large HDL | Triglycerides | 0.030 | 0.012 |
| 23536 | Triglycerides in Large LDL | Triglycerides | 0.097 | 0.025 |
| 23501 | Triglycerides in Large VLDL | Triglycerides | 0.167 | 0.095 |
| 23571 | Triglycerides in Medium HDL | Triglycerides | 0.054 | 0.019 |
| 23543 | Triglycerides in Medium LDL | Triglycerides | 0.033 | 0.010 |
| 23508 | Triglycerides in Medium VLDL | Triglycerides | 0.277 | 0.119 |
| 23578 | Triglycerides in Small HDL | Triglycerides | 0.052 | 0.018 |
| 23550 | Triglycerides in Small LDL | Triglycerides | 0.015 | 0.006 |
| 23515 | Triglycerides in Small VLDL | Triglycerides | 0.159 | 0.062 |
| 23557 | Triglycerides in Very Large HDL | Triglycerides | 0.007 | 0.003 |
| 23494 | Triglycerides in Very Large VLDL | Triglycerides | 0.109 | 0.082 |
| 23522 | Triglycerides in Very Small VLDL | Triglycerides | 0.069 | 0.021 |
| 23469 | Tyrosine | Amino acids | 0.063 | 0.014 |
| 23467 | Valine | Amino acids | 0.210 | 0.043 |
| 23474 | X3-Hydroxybutyrate | Ketone bodies | 0.059 | 0.060 |

Apo-A1, apolipoprotein A1; Apo-B, apolipoprotein B; DHA, docosahexaenoic acid; FA, fatty acids; Faw3, omega-3 fatty acids; Faw6, omega-6 fatty acids; HDL, high-density lipoproteins; IDL, intermediate-density lipoproteins; LA, linoleic acid; LDL, low-density lipoproteins; MUFA, monounsaturated fatty acids; PUFA, polyunsaturated fatty acids; SFA, saturated fatty acids; VLDL, very low-density lipoproteins

Table S5 Associations of clinical biomarkers with PC risk

| Variable* | HR (95% CI) * | *P-adj** |
| --- | --- | --- |
| Age | 2.48 (2.24, 2.75) | <0.001 |
| Sex | 1.22 (1.04, 1.43) | 0.031 |
| Ethnicity | 0.55 (0.37, 0.81) | -0.006 |
| Blood type | 0.80 (0.68, 0.94) | 0.017 |
| TDI | 1.24 (1.15, 1.33) | <0.001 |
| BMI | 1.30 (1.21, 1.40) | <0.001 |
| Height | 0.93 (0.83, 1.05) | 0.306 |
| Weight | 1.31 (1.20, 1.42) | <0.001 |
| Waist circumference | 1.38 (1.26, 1.50) | <0.001 |
| Smoking status | 1.42 (1.26, 1.60) | <0.001 |
| Drinking status | 0.94 (0.84, 1.05) | 0.340 |
| Sleep duration | 0.98 (0.91, 1.06) | 0.711 |
| Summed MET minutes | 1.06 (0.98, 1.14) | 0.179 |
| Diabetes mellitus | 1.59 (0.95, 2.66) | 0.118 |
| Chronic pancreatitis | 3.45 (0.49, 24.53) | 0.285 |
| Ulcerative colitis | 0.39 (0.06, 2.79) | 0.426 |
| Glucose | 1.13 (1.07, 1.18) | <0.001 |
| HbA1c | 1.18 (1.12, 1.24) | <0.001 |
| ALT | 1.10 (1.03, 1.17) | 0.007 |
| AST | 1.06 (1.01, 1.12) | 0.007 |
| ALP | 1.09 (1.03, 1.16) | 0.006 |
| GGT | 1.12 (1.06, 1.17) | <0.01 |
| Triglycerides | 1.13 (1.05, 1.21) | 0.003 |
| Cholesterol | 0.90 (0.83, 0.97) | 0.017 |
| TBIL | 0.95 (0.87, 1.04) | 0.382 |
| HDL | 0.87 (0.79, 0.95) | 0.006 |
| LDL | 0.91 (0.84, 0.99) | 0.038 |
| Neutrophil | 1.16 (1.08, 1.25) | <0.001 |
| Lymphocyte | 1.03 (0.99, 1.06) | 0.170 |
| WBC | 1.08 (1.05, 1.12) | <0.001 |
| PLT | 0.98 (0.90, 1.06) | 0.670 |

* HRs (per 1-SD increase) were adjusted for age and sex.

ALP, alkaline phosphatase; ALT, alanine aminotransferase; AST, aspartate aminotransferase; BMI, body mass index; GGT, gamma-glutamyl transferase; HbA1c, glycated hemoglobin; HDL, high-density lipoprotein; LDL, low-density lipoprotein; MET, metabolic equivalent of task; PLT, platelet; TDI, Townsend deprivation index; TBIL, total bilirubin; WBC, white blood cell

Table S6 Associations of metabolites with PC and results of sensitivity analysis

| Variable | Main model | | Excluding cases within first 2 years | | Using age as time scale | | Adjusting for diabetes mellitus | |
| --- | --- | --- | --- | --- | --- | --- | --- | --- |
|  | HR (95% CI)^1^ | *P-adj*^2^ | HR (95% CI) ^1^ | *P-adj*^2^ | HR (95% CI) ^1^ | *P-adj*^2^ | HR (95% CI) ^1^ | *P-adj*^2^ |
| Acetate | 1.01 (0.92, 1.10) | 0.879 | 1.03 (0.97, 1.10) | 0.353 | 1.03 (0.97, 1.09) | 0.475 | 1.03 (0.97, 1.09) | 0.495 |
| Acetoacetate | 1.04 (0.96, 1.12) | 0.408 | 1.06 (1.00, 1.12) | 0.102 | 1.05 (0.99, 1.11) | 0.129 | 1.05 (0.99, 1.11) | 0.142 |
| Acetone | 1.02 (0.94, 1.10) | 0.754 | 1.06 (1.00, 1.12) | 0.092 | 1.05 (0.99, 1.11) | 0.130 | 1.05 (0.99, 1.11) | 0.151 |
| Alanine | 1.00 (0.93, 1.09) | 0.954 | 1.00 (0.93, 1.07) | 0.920 | 1.00 (0.93, 1.07) | 0.908 | 0.99 (0.92, 1.06) | 0.749 |
| Albumin | 0.91 (0.84, 0.99) | 0.048 | 0.95 (0.89, 1.02) | 0.201 | 0.93 (0.88, 0.99) | 0.048 | 0.94 (0.88, 1.00) | 0.060 |
| ApoB/ApoA1 | 0.95 (0.87, 1.03) | 0.254 | 0.99 (0.92, 1.06) | 0.822 | 0.98 (0.92, 1.05) | 0.634 | 0.99 (0.92, 1.06) | 0.786 |
| Apolipoprotein A1 | 0.92 (0.84, 1.00) | 0.087 | 0.91 (0.84, 0.98) | 0.029 | 0.92 (0.85, 0.99) | 0.041 | 0.92 (0.86, 0.99) | 0.054 |
| Apolipoprotein B | 0.90 (0.83, 0.97) | 0.020 | 0.94 (0.88, 1.01) | 0.112 | 0.93 (0.87, 1.00) | 0.072 | 0.94 (0.88, 1.01) | 0.142 |
| Average Diameter for HDL Particles | 0.85 (0.78, 0.93) | 0.002 | 0.81 (0.75, 0.88) | <0.001 | 0.82 (0.76, 0.89) | <0.001 | 0.83 (0.76, 0.89) | <0.001 |
| Average Diameter for LDL Particles | 0.86 (0.79, 0.93) | 0.001 | 0.87 (0.82, 0.94) | 0.001 | 0.87 (0.82, 0.93) | <0.001 | 0.88 (0.82, 0.94) | <0.001 |
| Average Diameter for VLDL Particles | 1.17 (1.07, 1.27) | 0.001 | 1.18 (1.10, 1.27) | <0.001 | 1.18 (1.10, 1.27) | <0.001 | 1.18 (1.10, 1.27) | <0.001 |
| Cholesterol in Chylomicrons and Extremely Large VLDL | 1.14 (1.05, 1.23) | 0.003 | 1.14 (1.07, 1.22) | 0.001 | 1.15 (1.07, 1.22) | <0.001 | 1.15 (1.07, 1.22) | <0.001 |
| Cholesterol in IDL | 0.83 (0.77, 0.90) | <0.001 | 0.87 (0.81, 0.93) | <0.001 | 0.86 (0.80, 0.92) | <0.001 | 0.87 (0.81, 0.93) | <0.001 |
| Cholesterol in Large HDL | 0.82 (0.75, 0.90) | <0.001 | 0.80 (0.74, 0.87) | <0.001 | 0.80 (0.74, 0.87) | <0.001 | 0.81 (0.74, 0.87) | <0.001 |
| Cholesterol in Large LDL | 0.84 (0.78, 0.92) | <0.001 | 0.89 (0.83, 0.96) | 0.003 | 0.88 (0.83, 0.95) | 0.001 | 0.89 (0.83, 0.96) | 0.003 |
| Cholesterol in Large VLDL | 1.06 (0.97, 1.14) | 0.261 | 1.09 (1.01, 1.17) | 0.038 | 1.09 (1.02, 1.16) | 0.031 | 1.09 (1.02, 1.17) | 0.027 |
| Cholesterol in Medium HDL | 0.92 (0.84, 1.00) | 0.088 | 0.91 (0.84, 0.98) | 0.024 | 0.91 (0.85, 0.98) | 0.031 | 0.92 (0.85, 0.99) | 0.040 |
| Cholesterol in Medium LDL | 0.91 (0.84, 0.99) | 0.040 | 0.96 (0.89, 1.03) | 0.298 | 0.95 (0.89, 1.02) | 0.199 | 0.96 (0.90, 1.03) | 0.331 |
| Cholesterol in Medium VLDL | 0.88 (0.82, 0.96) | 0.007 | 0.92 (0.86, 0.99) | 0.034 | 0.91 (0.85, 0.98) | 0.017 | 0.92 (0.86, 0.99) | 0.041 |
| Cholesterol in Small HDL | 0.98 (0.91, 1.07) | 0.754 | 1.02 (0.95, 1.09) | 0.718 | 1.02 (0.95, 1.09) | 0.683 | 1.02 (0.95, 1.09) | 0.622 |
| Cholesterol in Small LDL | 0.90 (0.83, 0.97) | 0.020 | 0.95 (0.88, 1.01) | 0.160 | 0.94 (0.88, 1.00) | 0.091 | 0.95 (0.88, 1.01) | 0.160 |
| Cholesterol in Small VLDL | 0.96 (0.89, 1.04) | 0.408 | 1.00 (0.93, 1.07) | 0.917 | 1.00 (0.93, 1.06) | 0.899 | 1.00 (0.94, 1.07) | 0.948 |
| Cholesterol in Very Large HDL | 0.81 (0.73, 0.89) | <0.001 | 0.79 (0.73, 0.86) | <0.001 | 0.79 (0.73, 0.86) | <0.001 | 0.80 (0.74, 0.86) | <0.001 |
| Cholesterol in Very Large VLDL | 1.07 (0.99, 1.16) | 0.143 | 1.10 (1.02, 1.17) | 0.021 | 1.10 (1.02, 1.17) | 0.016 | 1.10 (1.03, 1.18) | 0.012 |
| Cholesterol in Very Small VLDL | 0.90 (0.83, 0.98) | 0.028 | 0.92 (0.86, 0.99) | 0.037 | 0.92 (0.86, 0.98) | 0.027 | 0.93 (0.86, 0.99) | 0.054 |
| Cholesteryl Esters in Chylomicrons and Extremely Large VLDL | 1.13 (1.04, 1.22) | 0.007 | 1.14 (1.06, 1.22) | 0.001 | 1.14 (1.07, 1.22) | <0.001 | 1.14 (1.07, 1.22) | <0.001 |
| Cholesteryl Esters in IDL | 0.83 (0.77, 0.90) | <0.001 | 0.87 (0.81, 0.93) | <0.001 | 0.86 (0.81, 0.92) | <0.001 | 0.87 (0.81, 0.93) | <0.001 |
| Cholesteryl Esters in Large HDL | 0.82 (0.75, 0.90) | <0.001 | 0.80 (0.74, 0.87) | <0.001 | 0.80 (0.74, 0.87) | <0.001 | 0.80 (0.74, 0.87) | <0.001 |
| Cholesteryl Esters in Large LDL | 0.85 (0.79, 0.93) | 0.001 | 0.90 (0.84, 0.97) | 0.008 | 0.89 (0.84, 0.96) | 0.003 | 0.90 (0.84, 0.97) | 0.008 |
| Cholesteryl Esters in Large VLDL | 1.02 (0.94, 1.11) | 0.689 | 1.05 (0.98, 1.13) | 0.200 | 1.05 (0.98, 1.13) | 0.185 | 1.06 (0.99, 1.13) | 0.148 |
| Cholesteryl Esters in Medium HDL | 0.92 (0.84, 1.01) | 0.100 | 0.91 (0.84, 0.98) | 0.027 | 0.92 (0.85, 0.99) | 0.034 | 0.92 (0.85, 0.99) | 0.043 |
| Cholesteryl Esters in Medium LDL | 0.93 (0.86, 1.01) | 0.148 | 0.98 (0.91, 1.05) | 0.644 | 0.98 (0.91, 1.04) | 0.536 | 0.99 (0.92, 1.05) | 0.716 |
| Cholesteryl Esters in Medium VLDL | 0.86 (0.79, 0.93) | 0.001 | 0.89 (0.83, 0.95) | 0.003 | 0.88 (0.82, 0.94) | 0.001 | 0.89 (0.83, 0.95) | 0.002 |
| Cholesteryl Esters in Small HDL | 0.99 (0.91, 1.07) | 0.843 | 1.02 (0.95, 1.09) | 0.669 | 1.02 (0.95, 1.09) | 0.654 | 1.02 (0.96, 1.09) | 0.598 |
| Cholesteryl Esters in Small LDL | 0.92 (0.85, 1.00) | 0.083 | 0.97 (0.90, 1.04) | 0.431 | 0.96 (0.90, 1.03) | 0.320 | 0.97 (0.91, 1.04) | 0.487 |
| Cholesteryl Esters in Small VLDL | 0.98 (0.90, 1.06) | 0.689 | 1.01 (0.94, 1.08) | 0.805 | 1.01 (0.95, 1.08) | 0.762 | 1.02 (0.95, 1.09) | 0.638 |
| Cholesteryl Esters in Very Large HDL | 0.80 (0.73, 0.88) | <0.001 | 0.79 (0.73, 0.86) | <0.001 | 0.79 (0.73, 0.85) | <0.001 | 0.79 (0.73, 0.86) | <0.001 |
| Cholesteryl Esters in Very Large VLDL | 1.04 (0.96, 1.13) | 0.434 | 1.07 (1.00, 1.15) | 0.086 | 1.07 (1.00, 1.15) | 0.072 | 1.08 (1.01, 1.15) | 0.055 |
| Cholesteryl Esters in Very Small VLDL | 0.88 (0.81, 0.96) | 0.007 | 0.90 (0.84, 0.97) | 0.008 | 0.90 (0.84, 0.96) | 0.004 | 0.91 (0.85, 0.97) | 0.012 |
| Citrate | 1.00 (0.92, 1.09) | 0.993 | 1.04 (0.97, 1.11) | 0.357 | 1.03 (0.97, 1.10) | 0.423 | 1.03 (0.96, 1.10) | 0.531 |
| Concentration of Chylomicrons and Extremely Large VLDL Particles | 1.15 (1.07, 1.24) | 0.001 | 1.14 (1.07, 1.22) | <0.001 | 1.15 (1.08, 1.22) | <0.001 | 1.14 (1.07, 1.22) | <0.001 |
| Concentration of IDL Particles | 0.88 (0.81, 0.95) | 0.006 | 0.92 (0.86, 0.99) | 0.046 | 0.92 (0.86, 0.98) | 0.021 | 0.93 (0.86, 0.99) | 0.048 |
| Concentration of Large HDL Particles | 0.83 (0.76, 0.91) | 0.001 | 0.81 (0.75, 0.88) | <0.001 | 0.82 (0.75, 0.88) | <0.001 | 0.82 (0.76, 0.89) | <0.001 |
| Concentration of Large LDL Particles | 0.88 (0.81, 0.96) | 0.007 | 0.92 (0.86, 0.99) | 0.044 | 0.91 (0.85, 0.98) | 0.021 | 0.92 (0.86, 0.99) | 0.044 |
| Concentration of Large VLDL Particles | 1.09 (1.01, 1.18) | 0.056 | 1.12 (1.04, 1.20) | 0.004 | 1.12 (1.05, 1.19) | 0.003 | 1.12 (1.05, 1.19) | 0.003 |
| Concentration of Medium HDL Particles | 0.94 (0.86, 1.03) | 0.248 | 0.93 (0.86, 1.00) | 0.093 | 0.94 (0.87, 1.01) | 0.129 | 0.94 (0.87, 1.01) | 0.148 |
| Concentration of Medium LDL Particles | 0.94 (0.86, 1.01) | 0.151 | 0.97 (0.91, 1.04) | 0.500 | 0.97 (0.91, 1.04) | 0.419 | 0.98 (0.91, 1.05) | 0.597 |
| Concentration of Medium VLDL Particles | 0.96 (0.89, 1.05) | 0.447 | 1.00 (0.94, 1.08) | 0.948 | 1.00 (0.93, 1.07) | 0.975 | 1.01 (0.94, 1.08) | 0.862 |
| Concentration of Small HDL Particles | 1.01 (0.93, 1.09) | 0.927 | 1.04 (0.97, 1.11) | 0.393 | 1.04 (0.97, 1.11) | 0.329 | 1.04 (0.98, 1.12) | 0.290 |
| Concentration of Small LDL Particles | 0.92 (0.85, 1.00) | 0.089 | 0.96 (0.90, 1.03) | 0.327 | 0.95 (0.89, 1.02) | 0.221 | 0.96 (0.90, 1.03) | 0.347 |
| Concentration of Small VLDL Particles | 1.03 (0.95, 1.11) | 0.614 | 1.06 (0.99, 1.14) | 0.148 | 1.06 (0.99, 1.14) | 0.117 | 1.07 (1.00, 1.14) | 0.100 |
| Concentration of Very Large HDL Particles | 0.82 (0.74, 0.90) | <0.001 | 0.81 (0.74, 0.88) | <0.001 | 0.81 (0.75, 0.87) | <0.001 | 0.81 (0.75, 0.88) | <0.001 |
| Concentration of Very Large VLDL Particles | 1.12 (1.04, 1.21) | 0.011 | 1.14 (1.06, 1.22) | 0.001 | 1.14 (1.07, 1.21) | <0.001 | 1.14 (1.06, 1.21) | <0.001 |
| Concentration of Very Small VLDL Particles | 0.96 (0.88, 1.04) | 0.341 | 0.98 (0.91, 1.05) | 0.633 | 0.98 (0.92, 1.05) | 0.634 | 0.99 (0.92, 1.06) | 0.770 |
| Creatinine | 1.00 (0.91, 1.10) | 0.972 | 1.03 (0.95, 1.11) | 0.599 | 1.02 (0.95, 1.11) | 0.633 | 1.02 (0.94, 1.10) | 0.696 |
| DHA/FA | 0.83 (0.78, 0.89) | <0.001 | 0.82 (0.78, 0.87) | <0.001 | 0.82 (0.78, 0.87) | <0.001 | 0.82 (0.77, 0.87) | <0.001 |
| Docosahexaenoic Acid | 0.83 (0.77, 0.91) | <0.001 | 0.83 (0.77, 0.90) | <0.001 | 0.83 (0.77, 0.89) | <0.001 | 0.83 (0.77, 0.89) | <0.001 |
| Faw3/FA | 0.86 (0.80, 0.93) | 0.001 | 0.85 (0.79, 0.91) | <0.001 | 0.84 (0.79, 0.90) | <0.001 | 0.84 (0.78, 0.89) | <0.001 |
| Faw6/FA | 0.83 (0.77, 0.90) | <0.001 | 0.84 (0.78, 0.90) | <0.001 | 0.84 (0.78, 0.89) | <0.001 | 0.84 (0.79, 0.90) | <0.001 |
| Free Cholesterol in Chylomicrons and Extremely Large VLDL | 1.15 (1.07, 1.24) | 0.001 | 1.14 (1.07, 1.22) | 0.001 | 1.15 (1.07, 1.22) | <0.001 | 1.14 (1.07, 1.22) | <0.001 |
| Free Cholesterol in IDL | 0.84 (0.78, 0.91) | <0.001 | 0.87 (0.81, 0.94) | 0.001 | 0.87 (0.81, 0.93) | <0.001 | 0.88 (0.82, 0.94) | 0.001 |
| Free Cholesterol in Large HDL | 0.83 (0.76, 0.91) | 0.001 | 0.81 (0.75, 0.88) | <0.001 | 0.81 (0.75, 0.88) | <0.001 | 0.82 (0.75, 0.89) | <0.001 |
| Free Cholesterol in Large LDL | 0.83 (0.77, 0.90) | <0.001 | 0.87 (0.81, 0.94) | 0.001 | 0.87 (0.81, 0.93) | <0.001 | 0.87 (0.82, 0.94) | <0.001 |
| Free Cholesterol in Large VLDL | 1.09 (1.00, 1.18) | 0.074 | 1.12 (1.04, 1.20) | 0.005 | 1.11 (1.04, 1.19) | 0.004 | 1.12 (1.04, 1.19) | 0.003 |
| Free Cholesterol in Medium HDL | 0.91 (0.84, 1.00) | 0.081 | 0.91 (0.84, 0.98) | 0.026 | 0.91 (0.85, 0.99) | 0.033 | 0.92 (0.85, 0.99) | 0.043 |
| Free Cholesterol in Medium LDL | 0.86 (0.79, 0.93) | 0.001 | 0.91 (0.85, 0.98) | 0.016 | 0.90 (0.84, 0.96) | 0.005 | 0.91 (0.85, 0.97) | 0.013 |
| Free Cholesterol in Medium VLDL | 0.93 (0.86, 1.01) | 0.137 | 0.97 (0.90, 1.04) | 0.469 | 0.97 (0.90, 1.03) | 0.367 | 0.97 (0.91, 1.04) | 0.531 |
| Free Cholesterol in Small HDL | 0.98 (0.90, 1.06) | 0.664 | 1.01 (0.94, 1.09) | 0.855 | 1.01 (0.95, 1.09) | 0.729 | 1.02 (0.95, 1.09) | 0.665 |
| Free Cholesterol in Small LDL | 0.85 (0.78, 0.92) | <0.001 | 0.90 (0.84, 0.96) | 0.006 | 0.89 (0.83, 0.95) | 0.001 | 0.89 (0.84, 0.96) | 0.003 |
| Free Cholesterol in Small VLDL | 0.93 (0.86, 1.01) | 0.124 | 0.97 (0.90, 1.04) | 0.445 | 0.97 (0.90, 1.03) | 0.367 | 0.97 (0.91, 1.04) | 0.531 |
| Free Cholesterol in Very Large HDL | 0.83 (0.76, 0.91) | <0.001 | 0.82 (0.76, 0.88) | <0.001 | 0.82 (0.76, 0.88) | <0.001 | 0.82 (0.76, 0.88) | <0.001 |
| Free Cholesterol in Very Large VLDL | 1.10 (1.02, 1.19) | 0.034 | 1.12 (1.04, 1.20) | 0.005 | 1.12 (1.05, 1.19) | 0.003 | 1.12 (1.05, 1.20) | 0.003 |
| Free Cholesterol in Very Small VLDL | 0.95 (0.88, 1.03) | 0.315 | 0.97 (0.91, 1.04) | 0.501 | 0.97 (0.91, 1.04) | 0.498 | 0.98 (0.92, 1.05) | 0.648 |
| Glucose | 1.18 (1.09, 1.27) | <0.001 | 1.13 (1.06, 1.21) | 0.001 | 1.17 (1.10, 1.24) | <0.001 | 1.16 (1.08, 1.23) | <0.001 |
| Glutamine | 0.94 (0.87, 1.02) | 0.182 | 0.98 (0.91, 1.05) | 0.668 | 0.96 (0.89, 1.02) | 0.234 | 0.96 (0.89, 1.03) | 0.271 |
| Glycine | 0.81 (0.74, 0.89) | <0.001 | 0.85 (0.79, 0.92) | <0.001 | 0.85 (0.78, 0.91) | <0.001 | 0.85 (0.79, 0.91) | <0.001 |
| Glycoprotein Acetyls | 1.18 (1.09, 1.28) | <0.001 | 1.22 (1.14, 1.31) | <0.001 | 1.24 (1.16, 1.33) | <0.001 | 1.23 (1.15, 1.32) | <0.001 |
| Histidine | 0.90 (0.82, 0.98) | 0.030 | 0.93 (0.87, 1.00) | 0.093 | 0.93 (0.86, 0.99) | 0.056 | 0.93 (0.86, 1.00) | 0.060 |
| Isoleucine | 1.03 (0.95, 1.11) | 0.556 | 1.07 (1.00, 1.14) | 0.066 | 1.06 (0.99, 1.13) | 0.129 | 1.05 (0.99, 1.12) | 0.151 |
| LA/FA | 0.86 (0.82, 0.89) | <0.001 | 0.85 (0.82, 0.89) | <0.001 | 0.85 (0.82, 0.89) | <0.001 | 0.86 (0.82, 0.90) | <0.001 |
| Lactate | 1.07 (0.98, 1.16) | 0.166 | 1.10 (1.03, 1.18) | 0.015 | 1.10 (1.02, 1.17) | 0.017 | 1.09 (1.02, 1.16) | 0.028 |
| Leucine | 1.02 (0.94, 1.11) | 0.713 | 1.06 (0.99, 1.14) | 0.107 | 1.05 (0.98, 1.12) | 0.199 | 1.05 (0.98, 1.12) | 0.233 |
| Linoleic Acid | 0.90 (0.83, 0.97) | 0.021 | 0.92 (0.86, 0.99) | 0.048 | 0.93 (0.87, 1.00) | 0.060 | 0.94 (0.88, 1.01) | 0.115 |
| Monounsaturated Fatty Acids | 1.11 (1.02, 1.20) | 0.033 | 1.13 (1.05, 1.21) | 0.003 | 1.13 (1.06, 1.21) | 0.001 | 1.13 (1.06, 1.21) | 0.001 |
| MUFA/FA | 1.23 (1.13, 1.34) | <0.001 | 1.24 (1.15, 1.33) | <0.001 | 1.25 (1.16, 1.33) | <0.001 | 1.24 (1.16, 1.33) | <0.001 |
| Omega-3 Fatty Acids | 0.90 (0.83, 0.98) | 0.028 | 0.89 (0.83, 0.96) | 0.005 | 0.89 (0.83, 0.95) | 0.002 | 0.89 (0.83, 0.95) | 0.002 |
| Omega-6 Fatty Acids | 0.91 (0.84, 0.99) | 0.049 | 0.94 (0.88, 1.01) | 0.152 | 0.95 (0.88, 1.02) | 0.169 | 0.96 (0.89, 1.03) | 0.267 |
| Phenylalanine | 1.06 (0.99, 1.13) | 0.150 | 1.07 (1.02, 1.13) | 0.011 | 1.07 (1.01, 1.13) | 0.029 | 1.07 (1.01, 1.12) | 0.036 |
| Phosphatidylcholines | 0.91 (0.84, 1.00) | 0.071 | 0.93 (0.86, 1.00) | 0.067 | 0.93 (0.87, 1.00) | 0.089 | 0.94 (0.87, 1.01) | 0.139 |
| Phosphoglycerides | 0.93 (0.86, 1.02) | 0.168 | 0.95 (0.88, 1.02) | 0.203 | 0.95 (0.89, 1.03) | 0.259 | 0.96 (0.89, 1.03) | 0.347 |
| Phospholipids in Chylomicrons and Extremely Large VLDL | 1.16 (1.07, 1.25) | 0.001 | 1.15 (1.07, 1.23) | <0.001 | 1.15 (1.08, 1.23) | <0.001 | 1.15 (1.08, 1.23) | <0.001 |
| Phospholipids in IDL | 0.85 (0.79, 0.93) | 0.001 | 0.88 (0.82, 0.95) | 0.002 | 0.88 (0.82, 0.94) | 0.001 | 0.89 (0.83, 0.95) | 0.002 |
| Phospholipids in Large HDL | 0.86 (0.78, 0.94) | 0.003 | 0.83 (0.77, 0.90) | <0.001 | 0.84 (0.77, 0.91) | <0.001 | 0.84 (0.78, 0.91) | <0.001 |
| Phospholipids in Large LDL | 0.87 (0.80, 0.94) | 0.001 | 0.91 (0.85, 0.98) | 0.016 | 0.90 (0.84, 0.97) | 0.006 | 0.91 (0.85, 0.98) | 0.017 |
| Phospholipids in Large VLDL | 1.10 (1.02, 1.19) | 0.036 | 1.13 (1.05, 1.21) | 0.002 | 1.13 (1.05, 1.21) | 0.001 | 1.13 (1.05, 1.21) | 0.001 |
| Phospholipids in Medium HDL | 0.99 (0.91, 1.08) | 0.861 | 0.97 (0.90, 1.05) | 0.533 | 0.98 (0.91, 1.06) | 0.699 | 0.98 (0.91, 1.06) | 0.716 |
| Phospholipids in Medium LDL | 0.91 (0.84, 0.99) | 0.038 | 0.96 (0.90, 1.03) | 0.353 | 0.95 (0.89, 1.02) | 0.230 | 0.96 (0.90, 1.03) | 0.363 |
| Phospholipids in Medium VLDL | 0.95 (0.88, 1.04) | 0.335 | 0.99 (0.93, 1.07) | 0.880 | 0.99 (0.93, 1.06) | 0.789 | 1.00 (0.93, 1.07) | 0.954 |
| Phospholipids in Small HDL | 1.07 (0.98, 1.16) | 0.167 | 1.08 (1.01, 1.16) | 0.061 | 1.09 (1.02, 1.17) | 0.029 | 1.09 (1.02, 1.17) | 0.028 |
| Phospholipids in Small LDL | 0.89 (0.82, 0.96) | 0.009 | 0.93 (0.87, 1.00) | 0.083 | 0.92 (0.86, 0.99) | 0.031 | 0.93 (0.87, 1.00) | 0.060 |
| Phospholipids in Small VLDL | 0.97 (0.89, 1.05) | 0.499 | 1.01 (0.94, 1.08) | 0.905 | 1.01 (0.94, 1.08) | 0.907 | 1.01 (0.95, 1.08) | 0.756 |
| Phospholipids in Very Large HDL | 0.83 (0.76, 0.92) | 0.001 | 0.81 (0.75, 0.88) | <0.001 | 0.81 (0.75, 0.88) | <0.001 | 0.82 (0.75, 0.88) | <0.001 |
| Phospholipids in Very Large VLDL | 1.11 (1.03, 1.21) | 0.018 | 1.13 (1.05, 1.21) | 0.002 | 1.13 (1.06, 1.21) | 0.001 | 1.13 (1.06, 1.21) | 0.001 |
| Phospholipids in Very Small VLDL | 0.99 (0.91, 1.07) | 0.831 | 1.00 (0.94, 1.08) | 0.928 | 1.01 (0.94, 1.08) | 0.848 | 1.02 (0.95, 1.09) | 0.725 |
| Polyunsaturated Fatty Acids | 0.90 (0.83, 0.97) | 0.021 | 0.92 (0.86, 0.99) | 0.046 | 0.93 (0.86, 0.99) | 0.046 | 0.93 (0.87, 1.00) | 0.075 |
| PUFA/FA | 0.80 (0.74, 0.86) | <0.001 | 0.80 (0.75, 0.85) | <0.001 | 0.79 (0.75, 0.85) | <0.001 | 0.80 (0.75, 0.85) | <0.001 |
| Pyruvate | 1.06 (0.99, 1.12) | 0.116 | 1.05 (0.99, 1.11) | 0.134 | 1.06 (1.00, 1.11) | 0.073 | 1.05 (1.00, 1.11) | 0.115 |
| Saturated Fatty Acids | 1.07 (0.98, 1.16) | 0.182 | 1.09 (1.01, 1.17) | 0.043 | 1.09 (1.02, 1.17) | 0.026 | 1.09 (1.02, 1.17) | 0.021 |
| SFA/FA | 1.16 (1.07, 1.26) | 0.001 | 1.15 (1.08, 1.23) | <0.001 | 1.15 (1.08, 1.23) | <0.001 | 1.15 (1.08, 1.23) | <0.001 |
| Sphingomyelins | 0.85 (0.78, 0.92) | 0.001 | 0.89 (0.83, 0.96) | 0.004 | 0.89 (0.83, 0.95) | 0.002 | 0.89 (0.83, 0.96) | 0.005 |
| Total Cholines | 0.90 (0.83, 0.98) | 0.038 | 0.92 (0.86, 0.99) | 0.052 | 0.93 (0.86, 1.00) | 0.062 | 0.94 (0.87, 1.01) | 0.105 |
| Total Fatty Acids | 1.03 (0.94, 1.11) | 0.637 | 1.05 (0.98, 1.13) | 0.241 | 1.06 (0.99, 1.13) | 0.172 | 1.06 (0.99, 1.14) | 0.148 |
| Total Lipids in Chylomicrons and Extremely Large VLDL | 1.15 (1.07, 1.25) | 0.001 | 1.14 (1.07, 1.23) | <0.001 | 1.15 (1.07, 1.23) | <0.001 | 1.15 (1.07, 1.22) | <0.001 |
| Total Lipids in IDL | 0.85 (0.78, 0.92) | <0.001 | 0.88 (0.82, 0.94) | 0.001 | 0.88 (0.82, 0.94) | <0.001 | 0.89 (0.83, 0.95) | 0.002 |
| Total Lipids in Large HDL | 0.84 (0.76, 0.92) | 0.001 | 0.81 (0.75, 0.88) | <0.001 | 0.82 (0.76, 0.88) | <0.001 | 0.82 (0.76, 0.89) | <0.001 |
| Total Lipids in Large LDL | 0.86 (0.79, 0.93) | 0.001 | 0.90 (0.84, 0.97) | 0.008 | 0.90 (0.84, 0.96) | 0.003 | 0.91 (0.85, 0.97) | 0.010 |
| Total Lipids in Large VLDL | 1.09 (1.00, 1.18) | 0.068 | 1.12 (1.04, 1.20) | 0.005 | 1.12 (1.04, 1.19) | 0.003 | 1.12 (1.04, 1.20) | 0.003 |
| Total Lipids in Medium HDL | 0.96 (0.88, 1.05) | 0.434 | 0.95 (0.88, 1.02) | 0.200 | 0.96 (0.89, 1.03) | 0.288 | 0.96 (0.89, 1.03) | 0.319 |
| Total Lipids in Medium LDL | 0.92 (0.84, 0.99) | 0.054 | 0.97 (0.90, 1.04) | 0.404 | 0.96 (0.90, 1.03) | 0.291 | 0.97 (0.91, 1.04) | 0.455 |
| Total Lipids in Medium VLDL | 0.98 (0.91, 1.07) | 0.753 | 1.03 (0.96, 1.10) | 0.575 | 1.02 (0.96, 1.09) | 0.589 | 1.03 (0.96, 1.10) | 0.483 |
| Total Lipids in Small HDL | 1.05 (0.97, 1.14) | 0.335 | 1.07 (1.00, 1.15) | 0.093 | 1.08 (1.01, 1.15) | 0.056 | 1.08 (1.01, 1.16) | 0.054 |
| Total Lipids in Small LDL | 0.90 (0.83, 0.98) | 0.030 | 0.95 (0.89, 1.02) | 0.217 | 0.94 (0.88, 1.01) | 0.129 | 0.95 (0.89, 1.02) | 0.221 |
| Total Lipids in Small VLDL | 1.02 (0.94, 1.11) | 0.720 | 1.05 (0.98, 1.13) | 0.200 | 1.06 (0.99, 1.13) | 0.152 | 1.06 (0.99, 1.14) | 0.130 |
| Total Lipids in Very Large HDL | 0.82 (0.75, 0.90) | <0.001 | 0.80 (0.74, 0.87) | <0.001 | 0.80 (0.74, 0.87) | <0.001 | 0.81 (0.75, 0.87) | <0.001 |
| Total Lipids in Very Large VLDL | 1.12 (1.04, 1.22) | 0.011 | 1.14 (1.06, 1.22) | 0.001 | 1.14 (1.07, 1.22) | <0.001 | 1.14 (1.07, 1.22) | 0.001 |
| Total Lipids in Very Small VLDL | 0.96 (0.89, 1.05) | 0.461 | 0.98 (0.91, 1.05) | 0.669 | 0.99 (0.92, 1.06) | 0.719 | 0.99 (0.93, 1.06) | 0.862 |
| Triglycerides in Chylomicrons and Extremely Large VLDL | 1.15 (1.07, 1.24) | 0.001 | 1.14 (1.07, 1.22) | 0.001 | 1.14 (1.07, 1.22) | <0.001 | 1.14 (1.07, 1.21) | <0.001 |
| Triglycerides in IDL | 1.09 (1.00, 1.18) | 0.078 | 1.10 (1.03, 1.18) | 0.015 | 1.11 (1.04, 1.19) | 0.004 | 1.11 (1.04, 1.19) | 0.005 |
| Triglycerides in Large HDL | 1.01 (0.93, 1.10) | 0.852 | 1.01 (0.94, 1.08) | 0.900 | 1.02 (0.95, 1.10) | 0.617 | 1.02 (0.95, 1.10) | 0.637 |
| Triglycerides in Large LDL | 1.08 (1.00, 1.17) | 0.087 | 1.11 (1.03, 1.18) | 0.010 | 1.12 (1.04, 1.19) | 0.003 | 1.12 (1.04, 1.19) | 0.003 |
| Triglycerides in Large VLDL | 1.10 (1.01, 1.19) | 0.047 | 1.12 (1.05, 1.20) | 0.003 | 1.12 (1.05, 1.20) | 0.002 | 1.12 (1.05, 1.20) | 0.002 |
| Triglycerides in Medium HDL | 1.13 (1.04, 1.22) | 0.007 | 1.13 (1.05, 1.21) | 0.002 | 1.14 (1.07, 1.22) | <0.001 | 1.14 (1.07, 1.22) | <0.001 |
| Triglycerides in Medium LDL | 1.09 (1.01, 1.18) | 0.065 | 1.11 (1.04, 1.19) | 0.005 | 1.12 (1.05, 1.20) | 0.002 | 1.12 (1.05, 1.20) | 0.002 |
| Triglycerides in Medium VLDL | 1.06 (0.98, 1.15) | 0.193 | 1.10 (1.02, 1.18) | 0.018 | 1.10 (1.03, 1.18) | 0.013 | 1.10 (1.03, 1.18) | 0.012 |
| Triglycerides in Small HDL | 1.16 (1.07, 1.26) | 0.001 | 1.18 (1.10, 1.26) | <0.001 | 1.19 (1.11, 1.27) | <0.001 | 1.18 (1.11, 1.26) | <0.001 |
| Triglycerides in Small LDL | 1.10 (1.02, 1.19) | 0.034 | 1.12 (1.04, 1.19) | 0.004 | 1.12 (1.05, 1.20) | 0.002 | 1.12 (1.05, 1.20) | 0.002 |
| Triglycerides in Small VLDL | 1.09 (1.01, 1.18) | 0.054 | 1.12 (1.04, 1.20) | 0.005 | 1.12 (1.05, 1.20) | 0.002 | 1.12 (1.05, 1.20) | 0.002 |
| Triglycerides in Very Large HDL | 1.02 (0.94, 1.10) | 0.760 | 1.02 (0.95, 1.10) | 0.599 | 1.03 (0.96, 1.11) | 0.423 | 1.03 (0.97, 1.11) | 0.436 |
| Triglycerides in Very Large VLDL | 1.14 (1.05, 1.23) | 0.004 | 1.15 (1.07, 1.23) | <0.001 | 1.15 (1.08, 1.23) | <0.001 | 1.15 (1.07, 1.23) | <0.001 |
| Triglycerides in Very Small VLDL | 1.11 (1.02, 1.20) | 0.028 | 1.12 (1.04, 1.20) | 0.004 | 1.13 (1.06, 1.21) | 0.001 | 1.13 (1.06, 1.20) | 0.001 |
| Tyrosine | 1.01 (0.93, 1.09) | 0.877 | 1.01 (0.94, 1.08) | 0.917 | 1.00 (0.93, 1.07) | 0.975 | 1.00 (0.93, 1.07) | 0.951 |
| Valine | 1.04 (0.96, 1.13) | 0.408 | 1.07 (1.00, 1.15) | 0.077 | 1.06 (0.99, 1.14) | 0.117 | 1.06 (0.99, 1.13) | 0.148 |
| X3-Hydroxybutyrate | 1.03 (0.95, 1.12) | 0.528 | 1.06 (1.00, 1.13) | 0.093 | 1.06 (0.99, 1.12) | 0.129 | 1.05 (0.99, 1.12) | 0.148 |

Apo-A1, apolipoprotein A1; Apo-B, apolipoprotein B; DHA, docosahexaenoic acid; FA, fatty acids; Faw3, omega-3 fatty acids; Faw6, omega-6 fatty acids; HDL, high-density lipoproteins; IDL, intermediate-density lipoproteins; LA, linoleic acid; LDL, low-density lipoproteins; MUFA, monounsaturated fatty acids; PUFA, polyunsaturated fatty acids; SFA, saturated fatty acids; VLDL, very low-density lipoproteins

^1^HRs (per 1-SD increase on the natural log scale) were adjusted for age and sex.

^2^*P*-values were adjusted for false discovery rate.

Table S7 Subgroup analyses stratified by age, sex, and BMI

| Variable | Age | | | Sex | | | BMI | | |
| --- | --- | --- | --- | --- | --- | --- | --- | --- | --- |
|  | Age < 55 (N = 66,076) | Age ≥ 55 (N = 91,159) | | Male (N = 73,721) | Female (N = 83,514) | | BMI < 25 (N = 56,768) | BMI ≥ 25 (N = 100,467) | |
|  | HR (95% CI) ^1^ | HR (95% CI) ^11^ | *P-interaction*^2^ | HR (95% CI) ^1^ | HR (95% CI) ^1^ | *P-interaction*^2^ | HR (95% CI) ^1^ | HR (95% CI) ^1^ | *P-interaction*^2^ |
| Acetate | 1.04 (0.89, 1.21) | 1.03 (0.96, 1.10) | 0.856 | 1.04 (0.97, 1.11) | 0.99 (0.85, 1.15) | 0.572 | 0.81 (0.54, 1.21) | 1.05 (0.99, 1.11) | 0.271 |
| Acetoacetate | 1.08 (0.94, 1.25) | 1.06 (1.00, 1.12) | 0.732 | 1.06 (0.97, 1.15) | 1.05 (0.96, 1.13) | 0.883 | 1.00 (0.88, 1.14) | 1.07 (1.00, 1.15) | 0.474 |
| Acetone | 1.02 (0.87, 1.20) | 1.06 (1.00, 1.12) | 0.642 | 1.08 (1.02, 1.16) | 0.99 (0.89, 1.10) | 0.156 | 1.06 (0.96, 1.17) | 1.05 (0.99, 1.12) | 0.737 |
| Alanine | 1.12 (0.94, 1.32) | 0.98 (0.91, 1.06) | 0.157 | 0.99 (0.90, 1.08) | 1.01 (0.91, 1.11) | 0.767 | 1.03 (0.90, 1.17) | 0.97 (0.90, 1.05) | 0.423 |
| Albumin | 0.80 (0.71, 0.91) | 0.93 (0.88, 0.99) | 0.048 | 1.00 (0.91, 1.09) | 0.87 (0.79, 0.95) | 0.036 | 1.05 (0.91, 1.21) | 0.92 (0.86, 0.98) | 0.473 |
| ApoB/ApoA1 | 1.08 (0.90, 1.31) | 0.93 (0.87, 1.01) | 0.096 | 0.90 (0.82, 0.99) | 1.08 (0.97, 1.19) | 0.012 | 1.02 (0.88, 1.17) | 0.93 (0.86, 1.01) | 0.008 |
| Apolipoprotein A1 | 0.86 (0.71, 1.05) | 0.94 (0.87, 1.02) | 0.301 | 1.02 (0.92, 1.13) | 0.82 (0.74, 0.92) | 0.005 | 0.99 (0.86, 1.15) | 0.93 (0.85, 1.02) | 0.285 |
| Apolipoprotein B | 1.00 (0.83, 1.21) | 0.90 (0.83, 0.96) | 0.219 | 0.90 (0.82, 0.99) | 0.97 (0.88, 1.07) | 0.319 | 1.02 (0.89, 1.18) | 0.89 (0.83, 0.97) | <0.001 |
| Average Diameter for HDL Particles | 0.80 (0.65, 0.98) | 0.85 (0.78, 0.93) | 0.421 | 0.91 (0.81, 1.02) | 0.76 (0.69, 0.85) | 0.030 | 0.91 (0.79, 1.05) | 0.85 (0.77, 0.94) | 0.523 |
| Average Diameter for LDL Particles | 0.89 (0.75, 1.05) | 0.86 (0.80, 0.92) | 0.910 | 0.88 (0.81, 0.96) | 0.86 (0.78, 0.96) | 0.738 | 1.04 (0.89, 1.21) | 0.86 (0.80, 0.93) | 0.028 |
| Average Diameter for VLDL Particles | 1.41 (1.17, 1.70) | 1.13 (1.05, 1.22) | 0.037 | 1.13 (1.03, 1.25) | 1.25 (1.12, 1.38) | 0.188 | 1.08 (0.93, 1.26) | 1.14 (1.04, 1.24) | 0.923 |
| Cholesterol in Chylomicrons and Extremely Large VLDL | 1.31 (1.12, 1.53) | 1.10 (1.02, 1.18) | 0.041 | 1.07 (0.98, 1.17) | 1.24 (1.13, 1.36) | 0.023 | 1.14 (0.97, 1.33) | 1.09 (1.01, 1.17) | 0.111 |
| Cholesterol in IDL | 0.85 (0.70, 1.04) | 0.84 (0.78, 0.90) | 0.993 | 0.88 (0.81, 0.97) | 0.83 (0.75, 0.92) | 0.340 | 0.95 (0.82, 1.10) | 0.85 (0.79, 0.92) | 0.001 |
| Cholesterol in Large HDL | 0.71 (0.57, 0.89) | 0.83 (0.76, 0.90) | 0.170 | 0.88 (0.78, 0.99) | 0.75 (0.68, 0.83) | 0.046 | 0.90 (0.78, 1.04) | 0.82 (0.74, 0.91) | 0.283 |
| Cholesterol in Large LDL | 0.85 (0.71, 1.03) | 0.86 (0.80, 0.93) | 0.893 | 0.89 (0.81, 0.97) | 0.87 (0.79, 0.97) | 0.787 | 0.98 (0.85, 1.13) | 0.86 (0.80, 0.93) | 0.001 |
| Cholesterol in Large VLDL | 1.26 (1.06, 1.50) | 1.04 (0.97, 1.12) | 0.034 | 1.02 (0.93, 1.13) | 1.15 (1.05, 1.26) | 0.086 | 1.12 (0.97, 1.29) | 1.01 (0.94, 1.10) | 0.007 |
| Cholesterol in Medium HDL | 0.82 (0.67, 1.00) | 0.94 (0.87, 1.02) | 0.154 | 1.02 (0.92, 1.13) | 0.82 (0.74, 0.91) | 0.004 | 0.98 (0.84, 1.13) | 0.94 (0.86, 1.02) | 0.630 |
| Cholesterol in Medium LDL | 0.96 (0.80, 1.15) | 0.92 (0.86, 0.99) | 0.580 | 0.93 (0.84, 1.02) | 0.98 (0.89, 1.08) | 0.422 | 1.02 (0.88, 1.17) | 0.91 (0.84, 0.98) | 0.001 |
| Cholesterol in Medium VLDL | 0.95 (0.78, 1.14) | 0.88 (0.82, 0.95) | 0.438 | 0.88 (0.80, 0.97) | 0.95 (0.86, 1.05) | 0.298 | 0.99 (0.86, 1.14) | 0.89 (0.82, 0.96) | 0.001 |
| Cholesterol in Small HDL | 0.93 (0.78, 1.12) | 1.01 (0.94, 1.09) | 0.407 | 1.04 (0.94, 1.14) | 0.99 (0.90, 1.10) | 0.507 | 1.04 (0.91, 1.19) | 0.99 (0.91, 1.07) | 0.271 |
| Cholesterol in Small LDL | 0.97 (0.81, 1.17) | 0.90 (0.84, 0.97) | 0.373 | 0.91 (0.83, 1.00) | 0.97 (0.87, 1.07) | 0.409 | 1.01 (0.87, 1.16) | 0.90 (0.83, 0.97) | 0.001 |
| Cholesterol in Small VLDL | 1.08 (0.91, 1.30) | 0.96 (0.89, 1.04) | 0.178 | 0.93 (0.84, 1.02) | 1.06 (0.96, 1.16) | 0.059 | 1.07 (0.94, 1.23) | 0.94 (0.87, 1.01) | <0.001 |
| Cholesterol in Very Large HDL | 0.72 (0.57, 0.91) | 0.81 (0.74, 0.88) | 0.282 | 0.83 (0.73, 0.94) | 0.77 (0.70, 0.85) | 0.420 | 0.92 (0.81, 1.05) | 0.78 (0.71, 0.87) | 0.016 |
| Cholesterol in Very Large VLDL | 1.27 (1.07, 1.50) | 1.05 (0.97, 1.13) | 0.031 | 1.03 (0.93, 1.13) | 1.17 (1.06, 1.28) | 0.062 | 1.11 (0.96, 1.29) | 1.03 (0.95, 1.11) | 0.015 |
| Cholesterol in Very Small VLDL | 0.99 (0.82, 1.20) | 0.90 (0.83, 0.97) | 0.401 | 0.91 (0.82, 1.00) | 0.92 (0.84, 1.02) | 0.806 | 1.03 (0.89, 1.18) | 0.89 (0.82, 0.96) | <0.001 |
| Cholesteryl Esters in Chylomicrons and Extremely Large VLDL | 1.28 (1.09, 1.50) | 1.10 (1.02, 1.18) | 0.079 | 1.06 (0.96, 1.16) | 1.25 (1.14, 1.37) | 0.013 | 1.14 (0.97, 1.33) | 1.08 (1.00, 1.17) | 0.089 |
| Cholesteryl Esters in IDL | 0.87 (0.71, 1.06) | 0.84 (0.78, 0.90) | 0.853 | 0.89 (0.81, 0.98) | 0.83 (0.75, 0.92) | 0.284 | 0.96 (0.83, 1.11) | 0.85 (0.79, 0.92) | 0.001 |
| Cholesteryl Esters in Large HDL | 0.70 (0.56, 0.88) | 0.83 (0.76, 0.90) | 0.135 | 0.88 (0.78, 0.99) | 0.75 (0.68, 0.83) | 0.060 | 0.90 (0.78, 1.03) | 0.82 (0.74, 0.91) | 0.302 |
| Cholesteryl Esters in Large LDL | 0.88 (0.73, 1.06) | 0.87 (0.81, 0.94) | 0.946 | 0.90 (0.82, 0.98) | 0.89 (0.80, 0.98) | 0.891 | 0.99 (0.86, 1.14) | 0.87 (0.81, 0.94) | <0.001 |
| Cholesteryl Esters in Large VLDL | 1.20 (1.01, 1.43) | 1.01 (0.94, 1.09) | 0.054 | 0.99 (0.89, 1.09) | 1.11 (1.02, 1.22) | 0.074 | 1.10 (0.96, 1.26) | 0.98 (0.91, 1.07) | 0.003 |
| Cholesteryl Esters in Medium HDL | 0.81 (0.67, 0.99) | 0.94 (0.87, 1.02) | 0.123 | 1.02 (0.92, 1.13) | 0.83 (0.74, 0.92) | 0.005 | 0.97 (0.84, 1.12) | 0.94 (0.86, 1.03) | 0.751 |
| Cholesteryl Esters in Medium LDL | 1.00 (0.83, 1.20) | 0.94 (0.88, 1.01) | 0.451 | 0.94 (0.86, 1.04) | 1.01 (0.91, 1.11) | 0.351 | 1.04 (0.90, 1.19) | 0.93 (0.86, 1.00) | 0.001 |
| Cholesteryl Esters in Medium VLDL | 0.87 (0.71, 1.05) | 0.85 (0.79, 0.92) | 0.907 | 0.86 (0.78, 0.94) | 0.90 (0.82, 1.00) | 0.489 | 0.95 (0.83, 1.10) | 0.87 (0.80, 0.94) | 0.002 |
| Cholesteryl Esters in Small HDL | 0.91 (0.76, 1.09) | 1.01 (0.94, 1.09) | 0.299 | 1.03 (0.94, 1.14) | 1.00 (0.91, 1.10) | 0.625 | 1.02 (0.89, 1.17) | 1.00 (0.92, 1.08) | 0.612 |
| Cholesteryl Esters in Small LDL | 1.01 (0.84, 1.21) | 0.92 (0.86, 0.99) | 0.270 | 0.93 (0.84, 1.02) | 1.00 (0.90, 1.10) | 0.301 | 1.03 (0.89, 1.19) | 0.92 (0.85, 0.99) | 0.001 |
| Cholesteryl Esters in Small VLDL | 1.11 (0.93, 1.33) | 0.98 (0.91, 1.06) | 0.163 | 0.94 (0.85, 1.03) | 1.08 (0.99, 1.18) | 0.040 | 1.08 (0.95, 1.24) | 0.95 (0.88, 1.03) | <0.001 |
| Cholesteryl Esters in Very Large HDL | 0.71 (0.56, 0.90) | 0.81 (0.74, 0.88) | 0.220 | 0.83 (0.73, 0.94) | 0.77 (0.69, 0.85) | 0.386 | 0.92 (0.81, 1.05) | 0.78 (0.70, 0.87) | 0.019 |
| Cholesteryl Esters in Very Large VLDL | 1.21 (1.01, 1.43) | 1.03 (0.95, 1.10) | 0.073 | 1.00 (0.90, 1.10) | 1.14 (1.04, 1.26) | 0.045 | 1.09 (0.95, 1.26) | 1.01 (0.93, 1.09) | 0.009 |
| Cholesteryl Esters in Very Small VLDL | 0.95 (0.78, 1.15) | 0.88 (0.81, 0.95) | 0.595 | 0.90 (0.81, 0.99) | 0.89 (0.81, 0.99) | 0.954 | 1.00 (0.86, 1.15) | 0.88 (0.81, 0.95) | <0.001 |
| Citrate | 1.05 (0.88, 1.26) | 1.07 (1.00, 1.15) | 0.778 | 1.08 (0.99, 1.19) | 0.98 (0.88, 1.07) | 0.149 | 0.93 (0.82, 1.07) | 1.09 (1.01, 1.17) | 0.154 |
| Concentration of Chylomicrons and Extremely Large VLDL Particles | 1.33 (1.16, 1.52) | 1.10 (1.02, 1.18) | 0.014 | 1.09 (1.00, 1.18) | 1.23 (1.12, 1.35) | 0.056 | 1.16 (0.98, 1.37) | 1.09 (1.01, 1.17) | 0.086 |
| Concentration of IDL Particles | 0.99 (0.82, 1.20) | 0.88 (0.82, 0.95) | 0.259 | 0.91 (0.83, 1.00) | 0.92 (0.83, 1.01) | 0.900 | 1.01 (0.87, 1.16) | 0.89 (0.82, 0.96) | <0.001 |
| Concentration of Large HDL Particles | 0.73 (0.58, 0.92) | 0.85 (0.78, 0.92) | 0.182 | 0.90 (0.79, 1.02) | 0.77 (0.69, 0.85) | 0.072 | 0.93 (0.82, 1.07) | 0.82 (0.74, 0.92) | 0.127 |
| Concentration of Large LDL Particles | 0.96 (0.80, 1.16) | 0.88 (0.82, 0.95) | 0.331 | 0.88 (0.80, 0.97) | 0.95 (0.86, 1.05) | 0.308 | 1.03 (0.89, 1.18) | 0.88 (0.81, 0.95) | <0.001 |
| Concentration of Large VLDL Particles | 1.30 (1.11, 1.53) | 1.07 (1.00, 1.15) | 0.025 | 1.06 (0.97, 1.16) | 1.18 (1.07, 1.30) | 0.113 | 1.16 (1.00, 1.35) | 1.04 (0.97, 1.13) | 0.010 |
| Concentration of Medium HDL Particles | 0.87 (0.71, 1.06) | 0.96 (0.89, 1.04) | 0.260 | 1.05 (0.94, 1.17) | 0.84 (0.76, 0.94) | 0.004 | 1.00 (0.87, 1.16) | 0.95 (0.87, 1.04) | 0.448 |
| Concentration of Medium LDL Particles | 0.99 (0.82, 1.18) | 0.94 (0.87, 1.01) | 0.525 | 0.93 (0.85, 1.03) | 1.00 (0.91, 1.11) | 0.319 | 1.01 (0.88, 1.16) | 0.93 (0.86, 1.01) | 0.003 |
| Concentration of Medium VLDL Particles | 1.11 (0.93, 1.32) | 0.96 (0.89, 1.03) | 0.101 | 0.95 (0.86, 1.05) | 1.05 (0.95, 1.15) | 0.165 | 1.07 (0.93, 1.23) | 0.94 (0.87, 1.02) | <0.001 |
| Concentration of Small HDL Particles | 0.97 (0.81, 1.17) | 1.03 (0.96, 1.11) | 0.585 | 1.06 (0.96, 1.16) | 1.02 (0.93, 1.12) | 0.617 | 1.07 (0.93, 1.22) | 1.00 (0.92, 1.08) | 0.182 |
| Concentration of Small LDL Particles | 1.03 (0.85, 1.23) | 0.92 (0.85, 0.99) | 0.207 | 0.92 (0.84, 1.02) | 0.98 (0.89, 1.08) | 0.372 | 1.02 (0.88, 1.17) | 0.91 (0.85, 0.99) | 0.001 |
| Concentration of Small VLDL Particles | 1.21 (1.03, 1.44) | 1.03 (0.96, 1.11) | 0.061 | 1.00 (0.91, 1.10) | 1.13 (1.02, 1.24) | 0.079 | 1.14 (0.99, 1.31) | 0.99 (0.91, 1.07) | 0.001 |
| Concentration of Very Large HDL Particles | 0.77 (0.61, 0.97) | 0.82 (0.76, 0.90) | 0.426 | 0.84 (0.74, 0.96) | 0.79 (0.71, 0.87) | 0.453 | 0.95 (0.84, 1.08) | 0.79 (0.71, 0.88) | 0.007 |
| Concentration of Very Large VLDL Particles | 1.33 (1.14, 1.55) | 1.09 (1.01, 1.17) | 0.019 | 1.08 (0.98, 1.18) | 1.21 (1.10, 1.33) | 0.091 | 1.18 (1.01, 1.37) | 1.06 (0.99, 1.15) | 0.017 |
| Concentration of Very Small VLDL Particles | 1.12 (0.93, 1.34) | 0.96 (0.89, 1.03) | 0.116 | 0.95 (0.86, 1.05) | 1.01 (0.91, 1.11) | 0.402 | 1.10 (0.96, 1.26) | 0.93 (0.86, 1.00) | <0.001 |
| Creatinine | 0.97 (0.76, 1.23) | 1.06 (0.98, 1.15) | 0.782 | 1.03 (0.94, 1.13) | 1.01 (0.88, 1.15) | 0.815 | 1.01 (0.85, 1.20) | 0.98 (0.89, 1.08) | 0.973 |
| DHA/FA | 0.78 (0.71, 0.86) | 0.87 (0.81, 0.93) | 0.052 | 0.83 (0.77, 0.89) | 0.81 (0.74, 0.88) | 0.659 | 0.85 (0.74, 0.98) | 0.83 (0.78, 0.89) | 0.495 |
| Docosahexaenoic Acid | 0.72 (0.58, 0.89) | 0.88 (0.82, 0.95) | 0.059 | 0.82 (0.74, 0.91) | 0.83 (0.75, 0.92) | 0.779 | 0.94 (0.82, 1.08) | 0.81 (0.74, 0.88) | 0.003 |
| Faw3/FA | 0.83 (0.71, 0.97) | 0.89 (0.83, 0.96) | 0.377 | 0.82 (0.75, 0.90) | 0.86 (0.78, 0.95) | 0.456 | 0.86 (0.76, 0.98) | 0.84 (0.77, 0.90) | 0.227 |
| Faw6/FA | 0.75 (0.64, 0.87) | 0.84 (0.78, 0.90) | 0.156 | 0.88 (0.81, 0.96) | 0.77 (0.70, 0.86) | 0.052 | 0.75 (0.64, 0.88) | 0.90 (0.84, 0.98) | <0.001 |
| Free Cholesterol in Chylomicrons and Extremely Large VLDL | 1.33 (1.15, 1.54) | 1.10 (1.02, 1.18) | 0.019 | 1.08 (0.99, 1.18) | 1.23 (1.12, 1.35) | 0.045 | 1.14 (0.97, 1.34) | 1.09 (1.01, 1.17) | 0.128 |
| Free Cholesterol in IDL | 0.83 (0.68, 1.01) | 0.85 (0.79, 0.92) | 0.657 | 0.88 (0.80, 0.96) | 0.85 (0.77, 0.94) | 0.655 | 0.96 (0.83, 1.11) | 0.86 (0.80, 0.93) | 0.001 |
| Free Cholesterol in Large HDL | 0.75 (0.60, 0.95) | 0.84 (0.77, 0.91) | 0.286 | 0.90 (0.79, 1.02) | 0.76 (0.69, 0.85) | 0.051 | 0.93 (0.81, 1.07) | 0.82 (0.74, 0.91) | 0.107 |
| Free Cholesterol in Large LDL | 0.80 (0.66, 0.97) | 0.85 (0.79, 0.91) | 0.537 | 0.88 (0.80, 0.96) | 0.85 (0.77, 0.94) | 0.614 | 0.96 (0.83, 1.10) | 0.85 (0.79, 0.92) | 0.001 |
| Free Cholesterol in Large VLDL | 1.30 (1.10, 1.53) | 1.07 (0.99, 1.15) | 0.028 | 1.05 (0.96, 1.16) | 1.18 (1.07, 1.30) | 0.089 | 1.15 (0.99, 1.33) | 1.04 (0.96, 1.13) | 0.012 |
| Free Cholesterol in Medium HDL | 0.86 (0.70, 1.06) | 0.94 (0.86, 1.01) | 0.329 | 1.03 (0.92, 1.15) | 0.82 (0.74, 0.91) | 0.003 | 1.00 (0.87, 1.15) | 0.92 (0.84, 1.01) | 0.220 |
| Free Cholesterol in Medium LDL | 0.86 (0.71, 1.04) | 0.88 (0.81, 0.94) | 0.912 | 0.89 (0.81, 0.98) | 0.91 (0.82, 1.01) | 0.761 | 0.98 (0.85, 1.13) | 0.88 (0.81, 0.95) | 0.002 |
| Free Cholesterol in Medium VLDL | 1.05 (0.88, 1.26) | 0.93 (0.86, 1.00) | 0.156 | 0.92 (0.84, 1.01) | 1.01 (0.92, 1.11) | 0.198 | 1.04 (0.91, 1.20) | 0.92 (0.85, 0.99) | <0.001 |
| Free Cholesterol in Small HDL | 1.01 (0.84, 1.21) | 1.01 (0.94, 1.09) | 0.925 | 1.05 (0.96, 1.16) | 0.97 (0.87, 1.07) | 0.238 | 1.11 (0.97, 1.28) | 0.97 (0.89, 1.05) | 0.007 |
| Free Cholesterol in Small LDL | 0.87 (0.72, 1.04) | 0.86 (0.80, 0.92) | 0.886 | 0.88 (0.80, 0.97) | 0.89 (0.81, 0.98) | 0.903 | 0.96 (0.83, 1.11) | 0.87 (0.81, 0.94) | 0.005 |
| Free Cholesterol in Small VLDL | 1.03 (0.86, 1.24) | 0.93 (0.87, 1.00) | 0.245 | 0.92 (0.83, 1.01) | 1.01 (0.92, 1.12) | 0.143 | 1.05 (0.92, 1.21) | 0.92 (0.85, 0.99) | <0.001 |
| Free Cholesterol in Very Large HDL | 0.80 (0.65, 1.00) | 0.82 (0.76, 0.89) | 0.641 | 0.84 (0.74, 0.95) | 0.80 (0.73, 0.88) | 0.586 | 0.94 (0.83, 1.06) | 0.81 (0.73, 0.89) | 0.009 |
| Free Cholesterol in Very Large VLDL | 1.32 (1.12, 1.54) | 1.07 (0.99, 1.15) | 0.016 | 1.05 (0.96, 1.16) | 1.19 (1.08, 1.31) | 0.078 | 1.14 (0.98, 1.32) | 1.05 (0.97, 1.13) | 0.020 |
| Free Cholesterol in Very Small VLDL | 1.09 (0.91, 1.31) | 0.95 (0.88, 1.02) | 0.166 | 0.94 (0.85, 1.04) | 1.00 (0.91, 1.10) | 0.386 | 1.10 (0.96, 1.26) | 0.92 (0.85, 0.99) | <0.001 |
| Glucose | 1.32 (1.14, 1.53) | 1.17 (1.09, 1.25) | 0.125 | 1.21 (1.12, 1.30) | 1.10 (0.99, 1.23) | 0.200 | 1.14 (0.99, 1.32) | 1.15 (1.07, 1.24) | 0.636 |
| Glutamine | 0.92 (0.78, 1.10) | 0.97 (0.90, 1.04) | 0.721 | 0.98 (0.89, 1.07) | 0.93 (0.84, 1.03) | 0.490 | 0.98 (0.85, 1.12) | 0.98 (0.90, 1.06) | 0.880 |
| Glycine | 0.76 (0.62, 0.92) | 0.85 (0.78, 0.92) | 0.211 | 0.87 (0.76, 0.99) | 0.84 (0.76, 0.92) | 0.654 | 0.92 (0.81, 1.06) | 0.85 (0.77, 0.93) | 0.348 |
| Glycoprotein Acetyls | 1.46 (1.24, 1.71) | 1.20 (1.12, 1.30) | 0.034 | 1.20 (1.10, 1.32) | 1.28 (1.16, 1.41) | 0.387 | 1.31 (1.14, 1.50) | 1.15 (1.06, 1.25) | 0.001 |
| Histidine | 0.85 (0.70, 1.02) | 0.92 (0.85, 0.99) | 0.501 | 0.97 (0.88, 1.07) | 0.88 (0.79, 0.98) | 0.154 | 1.00 (0.88, 1.14) | 0.90 (0.83, 0.98) | 0.297 |
| Isoleucine | 1.11 (0.94, 1.31) | 1.04 (0.97, 1.12) | 0.380 | 1.15 (1.06, 1.25) | 0.94 (0.85, 1.05) | 0.003 | 1.00 (0.87, 1.15) | 1.04 (0.97, 1.12) | 0.918 |
| LA/FA | 0.87 (0.82, 0.94) | 0.80 (0.75, 0.86) | 0.096 | 0.86 (0.79, 0.94) | 0.85 (0.81, 0.89) | 0.855 | 0.86 (0.80, 0.91) | 0.88 (0.82, 0.95) | 0.064 |
| Lactate | 1.13 (0.95, 1.35) | 1.09 (1.01, 1.17) | 0.681 | 1.10 (1.00, 1.20) | 1.09 (0.99, 1.20) | 0.912 | 1.07 (0.94, 1.23) | 1.09 (1.00, 1.17) | 0.966 |
| Leucine | 1.06 (0.89, 1.26) | 1.04 (0.96, 1.11) | 0.656 | 1.13 (1.04, 1.24) | 0.96 (0.87, 1.06) | 0.015 | 1.00 (0.87, 1.15) | 1.03 (0.95, 1.11) | 0.911 |
| Linoleic Acid | 1.06 (0.88, 1.28) | 0.89 (0.82, 0.95) | 0.078 | 0.94 (0.86, 1.03) | 0.91 (0.82, 1.01) | 0.621 | 0.97 (0.84, 1.12) | 0.92 (0.86, 1.00) | 0.020 |
| Monounsaturated Fatty Acids | 1.44 (1.23, 1.68) | 1.20 (1.11, 1.30) | 0.039 | 1.18 (1.07, 1.29) | 1.33 (1.20, 1.47) | 0.089 | 1.25 (1.08, 1.45) | 1.17 (1.08, 1.27) | 0.172 |
| MUFA/FA | 1.33 (1.13, 1.56) | 1.09 (1.01, 1.17) | 0.024 | 1.10 (1.00, 1.20) | 1.18 (1.06, 1.31) | 0.285 | 1.20 (1.03, 1.39) | 1.06 (0.98, 1.14) | 0.001 |
| Omega-3 Fatty Acids | 0.94 (0.78, 1.14) | 0.92 (0.85, 0.99) | 0.839 | 0.86 (0.78, 0.95) | 0.90 (0.82, 1.00) | 0.475 | 0.94 (0.82, 1.08) | 0.86 (0.79, 0.93) | 0.007 |
| Omega-6 Fatty Acids | 1.08 (0.90, 1.29) | 0.91 (0.84, 0.98) | 0.094 | 0.96 (0.88, 1.05) | 0.93 (0.83, 1.03) | 0.606 | 1.00 (0.87, 1.15) | 0.93 (0.86, 1.00) | 0.005 |
| Phenylalanine | 0.71 (0.61, 0.83) | 0.81 (0.76, 0.87) | 0.094 | 0.83 (0.77, 0.90) | 0.74 (0.67, 0.82) | 0.059 | 0.71 (0.61, 0.83) | 0.86 (0.79, 0.92) | 0.005 |
| Phosphatidylcholines | 1.02 (0.87, 1.19) | 1.10 (1.03, 1.18) | 0.394 | 1.08 (1.04, 1.13) | 1.01 (0.92, 1.12) | 0.228 | 0.96 (0.84, 1.11) | 1.07 (1.02, 1.13) | 0.313 |
| Phosphoglycerides | 1.01 (0.84, 1.22) | 0.92 (0.85, 1.00) | 0.538 | 1.00 (0.90, 1.10) | 0.86 (0.77, 0.95) | 0.043 | 1.05 (0.90, 1.22) | 0.91 (0.84, 0.99) | 0.004 |
| Phospholipids in Chylomicrons and Extremely Large VLDL | 1.03 (0.85, 1.24) | 0.94 (0.87, 1.02) | 0.517 | 1.01 (0.91, 1.11) | 0.89 (0.79, 0.99) | 0.087 | 1.06 (0.92, 1.23) | 0.93 (0.85, 1.01) | 0.003 |
| Phospholipids in IDL | 1.34 (1.16, 1.55) | 1.10 (1.03, 1.19) | 0.017 | 1.08 (0.99, 1.18) | 1.24 (1.13, 1.36) | 0.039 | 1.15 (0.97, 1.35) | 1.10 (1.02, 1.18) | 0.135 |
| Phospholipids in Large HDL | 0.89 (0.73, 1.09) | 0.86 (0.80, 0.93) | 0.859 | 0.89 (0.81, 0.98) | 0.87 (0.78, 0.96) | 0.712 | 0.99 (0.86, 1.14) | 0.86 (0.79, 0.93) | <0.001 |
| Phospholipids in Large LDL | 0.79 (0.64, 0.98) | 0.87 (0.80, 0.94) | 0.318 | 0.95 (0.84, 1.06) | 0.76 (0.69, 0.85) | 0.008 | 0.93 (0.81, 1.08) | 0.86 (0.78, 0.95) | 0.352 |
| Phospholipids in Large VLDL | 0.89 (0.73, 1.07) | 0.88 (0.81, 0.94) | 0.907 | 0.90 (0.82, 0.98) | 0.91 (0.82, 1.00) | 0.893 | 0.99 (0.86, 1.14) | 0.88 (0.81, 0.95) | <0.001 |
| Phospholipids in Medium HDL | 1.33 (1.13, 1.56) | 1.08 (1.00, 1.16) | 0.022 | 1.07 (0.97, 1.17) | 1.19 (1.09, 1.31) | 0.096 | 1.16 (1.00, 1.35) | 1.05 (0.97, 1.14) | 0.015 |
| Phospholipids in Medium LDL | 0.97 (0.80, 1.18) | 1.00 (0.92, 1.08) | 0.606 | 1.09 (0.99, 1.21) | 0.88 (0.79, 0.97) | 0.004 | 1.04 (0.90, 1.20) | 0.99 (0.90, 1.07) | 0.465 |
| Phospholipids in Medium VLDL | 0.99 (0.82, 1.19) | 0.92 (0.85, 0.99) | 0.368 | 0.93 (0.84, 1.02) | 0.98 (0.89, 1.09) | 0.398 | 1.04 (0.90, 1.19) | 0.91 (0.84, 0.98) | <0.001 |
| Phospholipids in Small HDL | 1.11 (0.93, 1.32) | 0.95 (0.88, 1.02) | 0.082 | 0.94 (0.86, 1.04) | 1.04 (0.94, 1.14) | 0.177 | 1.06 (0.92, 1.22) | 0.93 (0.86, 1.01) | <0.001 |
| Phospholipids in Small LDL | 1.09 (0.91, 1.30) | 1.08 (1.01, 1.17) | 0.971 | 1.12 (1.02, 1.23) | 1.05 (0.95, 1.16) | 0.317 | 1.11 (0.97, 1.27) | 1.04 (0.96, 1.13) | 0.231 |
| Phospholipids in Small VLDL | 1.03 (0.85, 1.23) | 0.88 (0.82, 0.95) | 0.100 | 0.91 (0.83, 1.00) | 0.94 (0.85, 1.03) | 0.680 | 1.03 (0.89, 1.19) | 0.88 (0.82, 0.95) | <0.001 |
| Phospholipids in Very Large HDL | 1.12 (0.94, 1.34) | 0.97 (0.90, 1.04) | 0.108 | 0.95 (0.86, 1.04) | 1.06 (0.97, 1.17) | 0.100 | 1.09 (0.95, 1.25) | 0.94 (0.87, 1.02) | <0.001 |
| Phospholipids in Very Large VLDL | 0.78 (0.63, 0.98) | 0.83 (0.76, 0.90) | 0.454 | 0.86 (0.76, 0.98) | 0.78 (0.71, 0.87) | 0.260 | 0.93 (0.82, 1.06) | 0.81 (0.73, 0.90) | 0.063 |
| Phospholipids in Very Small VLDL | 1.33 (1.14, 1.56) | 1.08 (1.00, 1.16) | 0.015 | 1.07 (0.97, 1.17) | 1.20 (1.09, 1.32) | 0.080 | 1.15 (0.99, 1.34) | 1.06 (0.98, 1.14) | 0.024 |
| Polyunsaturated Fatty Acids | 1.17 (0.99, 1.40) | 0.98 (0.91, 1.06) | 0.063 | 0.97 (0.88, 1.07) | 1.04 (0.94, 1.14) | 0.333 | 1.14 (1.00, 1.31) | 0.94 (0.87, 1.02) | <0.001 |
| PUFA/FA | 1.05 (0.88, 1.26) | 0.90 (0.83, 0.97) | 0.128 | 0.93 (0.85, 1.02) | 0.91 (0.82, 1.01) | 0.780 | 0.98 (0.85, 1.14) | 0.90 (0.83, 0.98) | 0.002 |
| Pyruvate | 1.06 (0.90, 1.23) | 1.06 (1.00, 1.12) | 0.873 | 1.04 (0.96, 1.12) | 1.07 (0.99, 1.16) | 0.623 | 1.07 (0.97, 1.18) | 1.04 (0.98, 1.11) | 0.671 |
| Saturated Fatty Acids | 1.15 (0.98, 1.36) | 1.16 (1.08, 1.25) | 0.978 | 1.15 (1.06, 1.26) | 1.15 (1.03, 1.28) | 0.944 | 1.21 (1.07, 1.38) | 1.10 (1.01, 1.19) | 0.138 |
| SFA/FA | 1.24 (1.05, 1.47) | 1.06 (0.98, 1.14) | 0.074 | 1.08 (0.99, 1.18) | 1.10 (0.99, 1.23) | 0.785 | 1.19 (1.02, 1.38) | 1.02 (0.94, 1.10) | <0.001 |
| Sphingomyelins | 0.80 (0.65, 0.97) | 0.89 (0.82, 0.96) | 0.218 | 0.92 (0.83, 1.01) | 0.85 (0.76, 0.94) | 0.264 | 1.04 (0.90, 1.20) | 0.86 (0.79, 0.94) | <0.001 |
| Total Cholines | 0.97 (0.80, 1.17) | 0.91 (0.85, 0.99) | 0.751 | 0.98 (0.89, 1.08) | 0.86 (0.77, 0.96) | 0.075 | 1.05 (0.90, 1.21) | 0.91 (0.83, 0.98) | 0.002 |
| Total Fatty Acids | 1.23 (1.04, 1.46) | 1.02 (0.94, 1.10) | 0.041 | 1.04 (0.95, 1.14) | 1.07 (0.96, 1.19) | 0.755 | 1.13 (0.97, 1.31) | 1.00 (0.92, 1.08) | 0.001 |
| Total Lipids in Chylomicrons and Extremely Large VLDL | 1.37 (1.17, 1.60) | 1.10 (1.02, 1.18) | 0.010 | 1.09 (1.00, 1.19) | 1.23 (1.11, 1.35) | 0.076 | 1.12 (0.95, 1.32) | 1.09 (1.01, 1.18) | 0.217 |
| Total Lipids in IDL | 0.90 (0.74, 1.09) | 0.85 (0.79, 0.92) | 0.748 | 0.89 (0.81, 0.98) | 0.85 (0.77, 0.94) | 0.493 | 0.98 (0.85, 1.14) | 0.86 (0.80, 0.93) | <0.001 |
| Total Lipids in Large HDL | 0.76 (0.62, 0.95) | 0.85 (0.78, 0.92) | 0.283 | 0.91 (0.81, 1.03) | 0.75 (0.67, 0.83) | 0.012 | 0.91 (0.79, 1.05) | 0.84 (0.76, 0.93) | 0.361 |
| Total Lipids in Large LDL | 0.88 (0.73, 1.06) | 0.87 (0.81, 0.94) | 0.917 | 0.90 (0.82, 0.98) | 0.89 (0.81, 0.99) | 0.938 | 0.99 (0.86, 1.15) | 0.87 (0.81, 0.94) | <0.001 |
| Total Lipids in Large VLDL | 1.32 (1.12, 1.57) | 1.07 (0.99, 1.15) | 0.020 | 1.07 (0.97, 1.17) | 1.17 (1.06, 1.29) | 0.179 | 1.13 (0.97, 1.30) | 1.05 (0.97, 1.13) | 0.033 |
| Total Lipids in Medium HDL | 0.92 (0.76, 1.11) | 0.98 (0.90, 1.06) | 0.407 | 1.06 (0.96, 1.17) | 0.85 (0.77, 0.95) | 0.004 | 1.01 (0.88, 1.17) | 0.97 (0.89, 1.05) | 0.504 |
| Total Lipids in Medium LDL | 0.99 (0.82, 1.18) | 0.93 (0.86, 1.00) | 0.432 | 0.93 (0.85, 1.02) | 0.99 (0.90, 1.10) | 0.375 | 1.03 (0.90, 1.19) | 0.91 (0.85, 0.99) | 0.001 |
| Total Lipids in Medium VLDL | 1.17 (0.98, 1.40) | 0.98 (0.91, 1.05) | 0.045 | 0.98 (0.89, 1.08) | 1.07 (0.97, 1.18) | 0.211 | 1.07 (0.93, 1.23) | 0.96 (0.89, 1.04) | 0.002 |
| Total Lipids in Small HDL | 1.06 (0.89, 1.27) | 1.07 (0.99, 1.15) | 0.948 | 1.10 (1.00, 1.21) | 1.05 (0.95, 1.16) | 0.496 | 1.10 (0.96, 1.26) | 1.03 (0.95, 1.12) | 0.202 |
| Total Lipids in Small LDL | 1.02 (0.85, 1.22) | 0.90 (0.84, 0.97) | 0.177 | 0.92 (0.84, 1.01) | 0.97 (0.88, 1.07) | 0.424 | 1.03 (0.89, 1.18) | 0.90 (0.84, 0.97) | <0.001 |
| Total Lipids in Small VLDL | 1.22 (1.03, 1.45) | 1.02 (0.95, 1.10) | 0.050 | 1.00 (0.90, 1.10) | 1.12 (1.02, 1.23) | 0.095 | 1.12 (0.98, 1.29) | 0.98 (0.91, 1.07) | 0.001 |
| Total Lipids in Very Large HDL | 0.77 (0.61, 0.96) | 0.82 (0.75, 0.89) | 0.438 | 0.85 (0.75, 0.96) | 0.77 (0.70, 0.86) | 0.291 | 0.92 (0.81, 1.05) | 0.80 (0.72, 0.89) | 0.038 |
| Total Lipids in Very Large VLDL | 1.35 (1.15, 1.59) | 1.09 (1.01, 1.17) | 0.016 | 1.08 (0.99, 1.19) | 1.21 (1.09, 1.33) | 0.114 | 1.15 (0.98, 1.34) | 1.07 (0.99, 1.16) | 0.043 |
| Total Lipids in Very Small VLDL | 1.14 (0.95, 1.36) | 0.96 (0.89, 1.03) | 0.084 | 0.96 (0.87, 1.06) | 1.01 (0.91, 1.11) | 0.463 | 1.11 (0.97, 1.27) | 0.93 (0.86, 1.01) | <0.001 |
| Triglycerides in Chylomicrons and Extremely Large VLDL | 1.36 (1.18, 1.57) | 1.09 (1.01, 1.17) | 0.006 | 1.09 (1.00, 1.19) | 1.21 (1.10, 1.34) | 0.110 | 1.12 (0.95, 1.33) | 1.09 (1.01, 1.17) | 0.234 |
| Triglycerides in IDL | 1.28 (1.10, 1.49) | 1.09 (1.01, 1.17) | 0.050 | 1.08 (0.98, 1.18) | 1.15 (1.04, 1.26) | 0.348 | 1.28 (1.12, 1.47) | 1.02 (0.94, 1.10) | <0.001 |
| Triglycerides in Large HDL | 1.12 (0.94, 1.33) | 1.04 (0.97, 1.12) | 0.544 | 1.07 (0.97, 1.18) | 0.97 (0.88, 1.07) | 0.207 | 1.09 (0.96, 1.25) | 1.00 (0.92, 1.09) | 0.056 |
| Triglycerides in Large LDL | 1.27 (1.09, 1.48) | 1.09 (1.02, 1.17) | 0.062 | 1.07 (0.98, 1.18) | 1.16 (1.05, 1.28) | 0.235 | 1.31 (1.14, 1.50) | 1.02 (0.94, 1.10) | <0.001 |
| Triglycerides in Large VLDL | 1.32 (1.12, 1.55) | 1.07 (1.00, 1.15) | 0.020 | 1.08 (0.99, 1.18) | 1.17 (1.06, 1.29) | 0.267 | 1.13 (0.97, 1.32) | 1.05 (0.98, 1.14) | 0.047 |
| Triglycerides in Medium HDL | 1.28 (1.09, 1.50) | 1.13 (1.05, 1.22) | 0.161 | 1.14 (1.04, 1.25) | 1.14 (1.04, 1.25) | 0.972 | 1.18 (1.03, 1.36) | 1.08 (1.00, 1.17) | 0.038 |
| Triglycerides in Medium LDL | 1.28 (1.10, 1.48) | 1.09 (1.02, 1.17) | 0.052 | 1.07 (0.98, 1.17) | 1.19 (1.08, 1.31) | 0.103 | 1.30 (1.13, 1.50) | 1.03 (0.96, 1.11) | <0.001 |
| Triglycerides in Medium VLDL | 1.28 (1.08, 1.51) | 1.06 (0.98, 1.14) | 0.034 | 1.06 (0.96, 1.16) | 1.14 (1.04, 1.26) | 0.258 | 1.13 (0.97, 1.31) | 1.03 (0.95, 1.11) | 0.014 |
| Triglycerides in Small HDL | 1.36 (1.16, 1.59) | 1.16 (1.08, 1.25) | 0.067 | 1.13 (1.03, 1.25) | 1.24 (1.13, 1.36) | 0.191 | 1.23 (1.07, 1.42) | 1.11 (1.02, 1.20) | 0.020 |
| Triglycerides in Small LDL | 1.30 (1.12, 1.50) | 1.08 (1.01, 1.16) | 0.023 | 1.07 (0.98, 1.17) | 1.19 (1.08, 1.32) | 0.092 | 1.23 (1.06, 1.44) | 1.05 (0.97, 1.13) | <0.001 |
| Triglycerides in Small VLDL | 1.32 (1.12, 1.55) | 1.10 (1.02, 1.18) | 0.034 | 1.07 (0.98, 1.18) | 1.18 (1.07, 1.30) | 0.174 | 1.19 (1.03, 1.37) | 1.05 (0.97, 1.13) | 0.004 |
| Triglycerides in Very Large HDL | 1.20 (1.03, 1.41) | 1.02 (0.95, 1.10) | 0.058 | 1.03 (0.94, 1.13) | 1.03 (0.93, 1.14) | 0.944 | 1.14 (0.99, 1.31) | 0.99 (0.92, 1.07) | 0.001 |
| Triglycerides in Very Large VLDL | 1.35 (1.16, 1.58) | 1.10 (1.03, 1.19) | 0.017 | 1.10 (1.01, 1.20) | 1.21 (1.10, 1.34) | 0.133 | 1.17 (1.00, 1.37) | 1.08 (1.00, 1.17) | 0.046 |
| Triglycerides in Very Small VLDL | 1.31 (1.13, 1.52) | 1.11 (1.03, 1.19) | 0.044 | 1.08 (0.99, 1.19) | 1.18 (1.07, 1.29) | 0.220 | 1.27 (1.10, 1.46) | 1.04 (0.96, 1.13) | <0.001 |
| Tyrosine | 1.10 (0.93, 1.30) | 1.01 (0.94, 1.08) | 0.338 | 1.09 (1.00, 1.20) | 0.90 (0.82, 1.00) | 0.007 | 0.97 (0.85, 1.12) | 0.97 (0.90, 1.05) | 0.520 |
| Valine | 1.09 (0.92, 1.30) | 1.05 (0.98, 1.13) | 0.559 | 1.14 (1.04, 1.25) | 0.98 (0.88, 1.08) | 0.024 | 0.98 (0.85, 1.13) | 1.04 (0.96, 1.12) | 0.839 |
| X3-Hydroxybutyrate | 1.10 (0.96, 1.26) | 1.06 (0.99, 1.14) | 0.686 | 1.09 (1.00, 1.18) | 1.02 (0.93, 1.12) | 0.329 | 1.02 (0.91, 1.15) | 1.08 (1.00, 1.16) | 0.625 |

Apo-A1, apolipoprotein A1; Apo-B, apolipoprotein B; DHA, docosahexaenoic acid; FA, fatty acids; Faw3, omega-3 fatty acids; Faw6, omega-6 fatty acids; HDL, high-density lipoproteins; IDL, intermediate-density lipoproteins; LA, linoleic acid; LDL, low-density lipoproteins; MUFA, monounsaturated fatty acids; PUFA, polyunsaturated fatty acids; SFA, saturated fatty acids; VLDL, very low-density lipoproteins

^1^HRs (per 1-SD increase on the natural log scale) were adjusted for age and sex.

^2^*P*-values were adjusted for false discovery rate.

Table S8 Metabolites selected by elastic net regression with non-zero coefficients

| Variable | β | Group |
| --- | --- | --- |
| Alanine | -0.013 | Amino acids |
| Glutamine | 0.167 | Amino acids |
| Glycine | -0.207 | Amino acids |
| Histidine | -0.110 | Amino acids |
| Isoleucine | 0.041 | Amino acids |
| Leucine | -0.196 | Amino acids |
| Phenylalanine | 0.041 | Amino acids |
| Tyrosine | 0.073 | Amino acids |
| ApoB /ApoA1 | -0.055 | Apolipoproteins |
| Cholesterol in Large LDL | -0.068 | Cholesterol |
| Cholesteryl Esters in Chylomicrons and Extremely Large VLDL | -0.196 | Cholesteryl esters |
| Cholesteryl Esters in IDL | -0.155 | Cholesteryl esters |
| Cholesteryl Esters in Large LDL | 0.000 | Cholesteryl esters |
| Sphingomyelins | -0.215 | Cholines |
| Total Cholines | -0.126 | Cholines |
| Omega 3 Fatty Acids | 0.053 | Fatty acids |
| Saturated Fatty Acids | 0.045 | Fatty acids |
| Total Fatty Acids | 0.172 | Fatty acids |
| SFA /FA | 0.041 | Fatty acids |
| PUFA /FA | -0.006 | Fatty acids |
| Faw6 /FA | -0.108 | Fatty acids |
| MUFA /FA | -0.050 | Fatty acids |
| LA /FA | -0.044 | Fatty acids |
| DHA /FA | -0.089 | Fatty acids |
| Albumin | -0.182 | Fluid balance |
| Creatinine | 0.023 | Fluid balance |
| Free Cholesterol in Very Large HDL | 0.077 | Free cholesterol |
| Free Cholesterol in Very Small VLDL | 0.434 | Free cholesterol |
| Glycoprotein Acetyls | 0.105 | GlycA |
| Citrate | 0.071 | Glycolysis |
| Glucose | 0.162 | Glycolysis |
| Lactate | 0.086 | Glycolysis |
| Pyruvate | 0.041 | Glycolysis |
| X3-Hydroxybutyrate | 0.013 | Ketone bodies |
| Acetate | -0.004 | Ketone bodies |
| Acetoacetate | -0.009 | Ketone bodies |
| Acetone | 0.055 | Ketone bodies |
| Concentration of IDL Particles | -0.004 | Lipoprotein particles & sizes |
| Concentration of Medium LDL Particles | 0.000 | Lipoprotein particles & sizes |
| Concentration of Small HDL Particles | 0.339 | Lipoprotein particles & sizes |
| Concentration of Very Large HDL Particles | 0.007 | Lipoprotein particles & sizes |
| Average Diameter for LDL Particles | -0.075 | Lipoprotein particles & sizes |
| Average Diameter for VLDL Particles | 0.234 | Lipoprotein particles & sizes |
| Phospholipids in Very Small VLDL | 0.134 | Phospholipids |
| Total Lipids in Chylomicrons and Extremely Large VLDL | -0.007 | Total lipids |
| Total Lipids in IDL | -0.096 | Total lipids |
| Total Lipids in Large LDL | -0.194 | Total lipids |
| Triglycerides in Chylomicrons and Extremely Large VLDL | -0.108 | Triglycerides |
| Triglycerides in Large LDL | 0.167 | Triglycerides |
| Triglycerides in Medium HDL | -0.236 | Triglycerides |
| Triglycerides in Medium VLDL | -0.080 | Triglycerides |
| Triglycerides in Small LDL | -0.232 | Triglycerides |

Apo-A1, apolipoprotein A1; Apo-B, apolipoprotein B; DHA, docosahexaenoic acid; FA, fatty acids; Faw3, omega-3 fatty acids; Faw6, omega-6 fatty acids; HDL, high-density lipoproteins; IDL, intermediate-density lipoproteins; LA, linoleic acid; LDL, low-density lipoproteins; MUFA, monounsaturated fatty acids; PUFA, polyunsaturated fatty acids; SFA, saturated fatty acids; VLDL, very low-density lipoproteins

Table S9 Relative contributions of clinical biomarkers, PRS, and the MRS in multivariable models

| Model | Variable | β | SE | HR (95% CI) | *P* |
| --- | --- | --- | --- | --- | --- |
| Age & Sex | Age | 0.909 | 0.053 | 2.48 (2.24, 2.75) | <0.001 |
|  | Sex | 0.196 | 0.081 | 1.22 (1.04, 1.43) | 0.015 |
| Age & Sex + clinical biomarkers | Waist circumference | 0.226 | 0.046 | 1.25 (1.15, 1.37) | <0.001 |
|  | HbA1c | 0.098 | 0.031 | 1.10 (1.04, 1.17) | 0.002 |
|  | Smoking | 0.267 | 0.063 | 1.31 (1.15, 1.48) | <0.001 |
|  | TDI | 0.154 | 0.039 | 1.17 (1.08, 1.26) | <0.001 |
|  | Neutrophil | 0.076 | 0.04 | 1.08 (1.00, 1.17) | 0.059 |
|  | GGT | 0.07 | 0.031 | 1.07 (1.01, 1.14) | 0.024 |
|  | ALP | 0.023 | 0.037 | 1.02 (0.95, 1.10) | 0.54 |
|  | Ethnicity | -0.498 | 0.2 | 0.61 (0.41, 0.90) | 0.013 |
|  | Age | 0.896 | 0.053 | 2.45 (2.21, 2.72) | <0.001 |
|  | Sex | -0.089 | 0.092 | 0.92 (0.76, 1.10) | 0.335 |
| Age & Sex + PRS | Age | 0.91 | 0.052 | 2.49 (2.24, 2.76) | <0.001 |
|  | Sex | 0.2 | 0.081 | 1.22 (1.04, 1.43) | 0.013 |
|  | PRS | 0.223 | 0.04 | 1.25 (1.16, 1.35) | <0.001 |
| Age & Sex + clinical biomarkers + PRS | Waist circumference | 0.226 | 0.046 | 1.25 (1.15, 1.37) | <0.001 |
|  | HbA1c | 0.096 | 0.031 | 1.10 (1.04, 1.17) | 0.002 |
|  | Smoking | 0.265 | 0.063 | 1.30 (1.15, 1.48) | <0.001 |
|  | TDI | 0.152 | 0.039 | 1.17 (1.08, 1.26) | <0.001 |
|  | Neutrophil | 0.079 | 0.04 | 1.08 (1.00, 1.17) | 0.048 |
|  | GGT | 0.061 | 0.031 | 1.06 (1.00, 1.13) | 0.049 |
|  | ALP | 0.043 | 0.035 | 1.04 (0.98, 1.12) | 0.214 |
|  | Ethnicity | -0.483 | 0.2 | 0.62 (0.42, 0.91) | 0.016 |
|  | Age | 0.896 | 0.053 | 2.45 (2.21, 2.72) | <0.001 |
|  | Sex | -0.076 | 0.092 | 0.93 (0.77, 1.11) | 0.411 |
|  | PRS | 0.227 | 0.04 | 1.26 (1.16, 1.36) | <0.001 |
| Age & Sex + MRS | Age | 0.837 | 0.053 | 2.31 (2.08, 2.56) | <0.001 |
|  | Sex | 0.116 | 0.082 | 1.12 (0.96, 1.32) | 0.153 |
|  | MRS | 0.339 | 0.039 | 1.40 (1.30, 1.52) | <0.001 |
| Age & Sex + clinical biomarkers + MRS | Waist circumference | 0.148 | 0.049 | 1.16 (1.06, 1.28) | 0.002 |
|  | HbA1c | 0.076 | 0.032 | 1.08 (1.01, 1.15) | 0.019 |
|  | Smoking | 0.244 | 0.063 | 1.28 (1.13, 1.44) | <0.001 |
|  | TDI | 0.151 | 0.039 | 1.16 (1.08, 1.26) | <0.001 |
|  | Neutrophil | 0.039 | 0.041 | 1.04 (0.96, 1.13) | 0.334 |
|  | GGT | 0.058 | 0.032 | 1.06 (1.00, 1.13) | 0.069 |
|  | ALP | 0.018 | 0.037 | 1.02 (0.95, 1.10) | 0.635 |
|  | Ethnicity | -0.551 | 0.2 | 0.58 (0.39, 0.85) | 0.006 |
|  | Age | 0.859 | 0.054 | 2.36 (2.12, 2.62) | <0.001 |
|  | Sex | -0.059 | 0.093 | 0.94 (0.79, 1.13) | 0.526 |
|  | MRS | 0.22 | 0.044 | 1.25 (1.14, 1.36) | <0.001 |
| Age & Sex + PRS + MRS | Age | 0.838 | 0.053 | 2.31 (2.08, 2.57) | <0.001 |
|  | Sex | 0.121 | 0.082 | 1.13 (0.96, 1.32) | 0.139 |
|  | MRS | 0.338 | 0.039 | 1.40 (1.30, 1.51) | <0.001 |
|  | PRS | 0.222 | 0.04 | 1.25 (1.16, 1.35) | <0.001 |
| Age & Sex + clinical biomarkers + PRS + MRS | Waist circumference | 0.15 | 0.049 | 1.16 (1.06, 1.28) | 0.002 |
|  | HbA1c | 0.075 | 0.033 | 1.08 (1.01, 1.15) | 0.022 |
|  | Smoking | 0.242 | 0.063 | 1.27 (1.13, 1.44) | <0.001 |
|  | TDI | 0.149 | 0.039 | 1.16 (1.08, 1.25) | <0.001 |
|  | Neutrophil | 0.043 | 0.041 | 1.04 (0.96, 1.13) | 0.292 |
|  | GGT | 0.05 | 0.032 | 1.05 (0.99, 1.12) | 0.118 |
|  | ALP | 0.038 | 0.035 | 1.04 (0.97, 1.11) | 0.28 |
|  | Ethnicity | -0.535 | 0.2 | 0.59 (0.40, 0.87) | 0.008 |
|  | Age | 0.859 | 0.054 | 2.36 (2.13, 2.62) | <0.001 |
|  | Sex | -0.046 | 0.093 | 0.96 (0.80, 1.15) | 0.619 |
|  | MRS | 0.216 | 0.044 | 1.24 (1.14, 1.35) | <0.001 |
|  | PRS | 0.225 | 0.04 | 1.25 (1.16, 1.35) | <0.001 |

ALP, alkaline phosphatase; GGT, gamma-glutamyl transferase; HbA1c, glycated hemoglobin; MRS, metabolomic risk score; PRS, polygenic risk score; SE, standard error; TDI, Townsend deprivation index

Table S10 Model performance in the training set

|  | C-index | 1-year AUC | 3-year AUC | 5-year AUC | 10 year AUC |
| --- | --- | --- | --- | --- | --- |
| Age & Sex | 0.720 (0.700 - 0.739) | 0.748 (0.633 - 0.840) | 0.717 (0.659 - 0.776) | 0.730 (0.688 - 0.775) | 0.720 (0.693 - 0.744) |
| Age & Sex + clinical biomarkers | 0.747 (0.728 - 0.765) | 0.820 (0.729 - 0.893) | 0.769 (0.721 - 0.817) | 0.763 (0.727 - 0.793) | 0.752 (0.732 - 0.771) |
| Age & Sex + PRS | 0.727 (0.708 - 0.746) | 0.769 (0.657 - 0.864) | 0.733 (0.674 - 0.792) | 0.741 (0.700 - 0.782) | 0.727 (0.703 - 0.747) |
| Age & Sex + clinical biomarkers + PRS | 0.753 (0.735 - 0.771) | 0.833 (0.764 - 0.890) | 0.787 (0.734 - 0.826) | 0.776 (0.741 - 0.803) | 0.757 (0.732 - 0.778) |
| Age & Sex + MRS | 0.738 (0.719 - 0.756) | 0.745 (0.640 - 0.836) | 0.741 (0.692 - 0.793) | 0.750 (0.718 - 0.786) | 0.741 (0.721 - 0.765) |
| Age & Sex + clinical biomarkers + MRS | 0.751 (0.733 - 0.770) | 0.808 (0.751 - 0.864) | 0.771 (0.727 - 0.817) | 0.768 (0.726 - 0.799) | 0.759 (0.742 - 0.781) |
| Age & Sex + PRS + MRS | 0.744 (0.726 - 0.763) | 0.775 (0.678 - 0.858) | 0.762 (0.704 - 0.822) | 0.763 (0.719 - 0.799) | 0.749 (0.726 - 0.771) |
| Age & Sex + clinical biomarkers + PRS + MRS | 0.760 (0.742 - 0.779) | 0.838 (0.757 - 0.911) | 0.789 (0.745 - 0.835) | 0.783 (0.750 - 0.815) | 0.765 (0.747 - 0.786) |

AUC, area under the curve; MRS, metabolomic risk score; PRS, polygenic risk score

Table S11 Model performance after excluding early events

| Model | C-index | 5-year AUC | 10-year AUC |
| --- | --- | --- | --- |
| Age & Sex | 0.715 (0.655-0.776) | 0.721 (0.655-0.782) | 0.729 (0.682-0.768) |
| Age & Sex + clinical biomarkers | 0.751 (0.693-0.810) | 0.745 (0.673-0.807) | 0.763 (0.722-0.797) |
| Age & Sex + PRS | 0.731 (0.672-0.790) | 0.711 (0.648-0.772) | 0.738 (0.705-0.776) |
| Age & Sex + clinical biomarkers + PRS | 0.765 (0.708-0.822) | 0.748 (0.671-0.825) | 0.775 (0.735-0.813) |
| Age & Sex + MRS | 0.726 (0.668-0.785) | 0.728 (0.676-0.796) | 0.738 (0.704-0.771) |
| Age & Sex + clinical biomarkers + MRS | 0.751 (0.694-0.808) | 0.736 (0.679-0.795) | 0.763 (0.732-0.801) |
| Age & Sex + PRS + MRS | 0.740 (0.682-0.798) | 0.718 (0.653-0.788) | 0.748 (0.711-0.793) |
| Age & Sex + clinical biomarkers + PRS + MRS | 0.764 (0.708-0.820) | 0.744 (0.677-0.824) | 0.776 (0.733-0.817) |

AUC, area under the curve; MRS, metabolomic risk score; PRS, polygenic risk score

Table S12 Calibration performance of the prediction models.

| Model | Intercept | Slope | R^2^ |
| --- | --- | --- | --- |
| Age & Sex | 0.000 | 1.094 | 0.899 |
| Age & Sex + clinical biomarkers | 0.000 | 1.020 | 0.963 |
| Age & Sex + PRS | 0.000 | 1.012 | 0.965 |
| Age & Sex + clinical biomarkers + PRS | 0.000 | 1.021 | 0.958 |
| Age & Sex + MRS | 0.000 | 0.962 | 0.915 |
| Age & Sex + clinical biomarkers + MRS | 0.000 | 1.023 | 0.975 |
| Age & Sex + PRS + MRS | 0.000 | 0.960 | 0.972 |
| Age & Sex + clinical biomarkers + PRS + MRS | 0.000 | 1.014 | 0.972 |

MRS, metabolomic risk score; PRS, polygenic risk score

Table S13 Continuous NRI and IDI between models and age & sex base model on the validation set.

| Model | NRI | *P* | IDI | *P* |
| --- | --- | --- | --- | --- |
| Age & Sex + clinical biomarkers | 0.196 (0.025, 0.356) | <0.001 | 0.001 (0.000, 0.003) | <0.001 |
| Age & Sex + PRS | 0.059 (-0.117, 0.246) | 0.627 | 0.000 (0.000, 0.001) | 0.219 |
| Age & Sex + clinical biomarkers + PRS | 0.189 (0.057, 0.327) | 0.010 | 0.002 (0.001, 0.004) | <0.001 |
| Age & Sex + MRS | 0.186 (-0.005, 0.293) | 0.070 | 0.000 (0.000, 0.001) | 0.119 |
| Age & Sex + clinical biomarkers + MRS | 0.260 (0.058, 0.367) | <0.001 | 0.001 (0.000, 0.003) | <0.001 |
| Age & Sex + PRS + MRS | 0.113 (-0.019, 0.300) | 0.100 | 0.001 (0.000, 0.002) | 0.010 |
| Age & Sex + clinical biomarkers + PRS + MRS | 0.223 (0.052, 0.354) | <0.001 | 0.002 (0.001, 0.006) | <0.001 |

IDI, integrated discrimination index; MRS, metabolomic risk score; NRI, net reclassification index; PRS, polygenic risk score

Table S14 Cutoff values for risk stratification

| Model | Cutoff |
| --- | --- |
| Age & Sex | 0.686 |
| Age & Sex + clinical biomarkers | 0.151 |
| Age & Sex + PRS | 0.623 |
| Age & Sex + clinical biomarkers + PRS | 0.286 |
| Age & Sex + MRS | 0.855 |
| Age & Sex + clinical biomarkers + MRS | 0.116 |
| Age & Sex + PRS + MRS | 0.849 |
| Age & Sex + clinical biomarkers + PRS + MRS | 0.491 |

MRS, metabolomic risk score; NRI, net reclassification index; PRS, polygenic risk score

Figure S1 Associations between biomarkers and PC risk in the training set, validation set, and total cohort

Figure S2 Associations between 143 individual metabolites and PC risk, excluding PC diagnoses within the first two years of follow-up

Figure S3 Associations between 143 individual metabolites and PC risk using age as the time scale

Figure S4 Associations between 143 individual metabolites and PC risk adjusted for diabetes

Figure S5 Associations between 143 individual metabolites and PC risk stratified by age

Figure S6 Associations between 143 individual metabolites and PC risk stratified by sex

Figure S7 Associations between 143 individual metabolites and PC risk stratified by BMI

Figure S8 Best-fit Elastic Net cross-validation plot and solution path. (**A**) The cross-validation plot identifies the optimal parameters that minimize the cross-validation error, with α = 0.5 and λ = 0.00375857209793828. (**B**) The solution path offers a concise overview of all optimal solutions within the model

Figure S9 Cumulative incidence curves for risk stratification across models
